# A digital twin-based comparative reinforcement learning framework for personalized behavioral recommendation

**DOI:** 10.3389/frai.2026.1834771

**Published:** 2026-06-25

**Authors:** Ayan Chatterjee, Nurilla Avazov

**Affiliations:** 1 Department of Digital Technology, Norwegian Institute for Air Research, Kjeller, Norway; 2 Inland School of Business and Social Sciences, University of Inland Norway, Lillehammer, Norway

**Keywords:** behavioral and contextual data, digital twin, DQN, recommendation generation, reinforcement learning, simulation

## Abstract

Promoting healthy lifestyle behaviors such as physical activity, sleep, diet quality, stress management, hydration, and healthy habits requires adaptive systems capable of responding dynamically to changing behavioral and environmental conditions. However, the development and evaluation of personalized recommendation systems are challenged by fragmented observational data, privacy constraints, delayed feedback, and ethical limitations associated with long-term human experimentation. To address these challenges, this study proposes a digital twin-driven reinforcement learning framework for generating personalized behavioral recommendations in a fully simulated and statistically validated environment. The proposed framework formulates personalized behavioral recommendation as a stochastic Markov Decision Process (MDP) incorporating adherence uncertainty, behavioral drift, environmental modulation, and engagement dynamics. Synthetic longitudinal behavioral trajectories are generated through a digital twin simulator that models demographic heterogeneity, lifestyle behaviors, contextual variables, and variability in policy adherence over time. The optimization objective is defined through an effective reward formulation that balances behavioral compliance gains against penalties associated with health and environmental constraint violations. This study implements several reinforcement learning (RL) paradigms under simulated conditions, such as multi-armed bandits, table-based Q-learning, State-Action-Reward-State-Action (SARSA), function approximation-based temporal difference (TD) learning, and deep Q-learning network (DQN). The results demonstrate that richer state representations and context-dependent action dynamics are necessary for higher-capacity reinforcement learning models to consistently outperform simpler baselines. Furthermore, this study provides a reproducible method for comparing learning dynamics, performance, and computational cost in digital twin-based recommender systems. The framework additionally supports privacy-preserving experimentation through the exclusive use of synthetic behavioral data and locally controlled simulation environments.

## 1 Introduction

Personalized behavioral recommendations have emerged as a promising paradigm for supporting long-term lifestyle improvements in areas such as physical activity, sleep hygiene, stress management, and nutrition (Akker et al., 2014; Chatterjee et al., 2021a,b; Rabbi et al., 2015). These issues are inherently sequential, uncertain, and multi-dimensional, making them well-suited for reinforcement learning models. However, the practical application of reinforcement learning systems in health and behavior change is limited by ethical concerns, limited observability, feedback delays, and the high costs of research involving human subjects (Jacob et al., 2025; Peels et al., 2014; Chatterjee et al., 2021c, 2022a,b). Digital twin technology bridges the methodological gap between theoretical reinforcement learning and its practical applications (Vlaev and Dolan, 2015; Cheng et al., 2024). By creating simulated, controllable replicas of individuals and their environments, digital twins enable large-scale experiments, algorithm comparisons, and sensitivity analyses under well-defined assumptions (Vlaev and Dolan, 2015; Cheng et al., 2024). Despite growing interest in digital twins, existing research often oversimplifies behavioral dynamics, effects, or evaluation schemes, making it difficult to assess when and why more advanced reinforcement learning methods are needed.

This study bridges these gaps by proposing a digital twin model that explicitly simulates behavioral deficiencies, adherence uncertainty, environmental adaptation, and participation dynamics, and evaluates multiple reinforcement learning paradigms under the same simulation conditions. The proposed simulation environment can enable us to test hypotheses, evaluate strategies, and train decision-making processes before actual deployment. It may enable safe research behavioral interventions to avoid health risks, quick iterations using multiple virtual agents (e.g., users with different profiles), stress-testing under extreme or unusual scenarios (e.g., dropout, misreporting, habit shocks), and quantitative comparison across multiple reinforcement learning paradigms, including multi-armed bandits, Q-learning (Clifton and Laber, 2020), SARSA (Wang et al., 2013), TD learning (Ueno et al., 2011), and deep Q-networks (DQN) (Fan et al., 2020). The simulation environment can ensure that reinforcement learning agents can continuously learn from behavioral patterns, improve their reward models (Yu et al., 2021; Frommeyer et al., 2025), and dynamically adapt to World Health Organization (WHO) guidelines or other relevant compliance frameworks. Insights gained from recent research advances highlight the need for a robust, adaptive, and simulation-centric framework for behavioral recommendation. Our approach builds on the proven utility of digital twins in capturing dynamically changing health states with high-fidelity (Katsoulakis et al., 2024; Rivera et al., 2019; Francis et al., 2024) and the effectiveness of reinforcement learning in facilitating personalized policy learning under deterministic and uncertain constraints in real-time. The work is guided by the following research questions:

- **RQ1:** To what extent do different reinforcement learning paradigms differ in performance under identical simulated behavioral dynamics?
- **RQ2:** To what extent are learned reinforcement learning policies sensitive to adherence uncertainty, environmental variability, and behavioral drift?
- **RQ3:** Can digital twin simulation environments support statistically robust comparison of reinforcement learning-based recommendation strategies?

**Figure 1:**
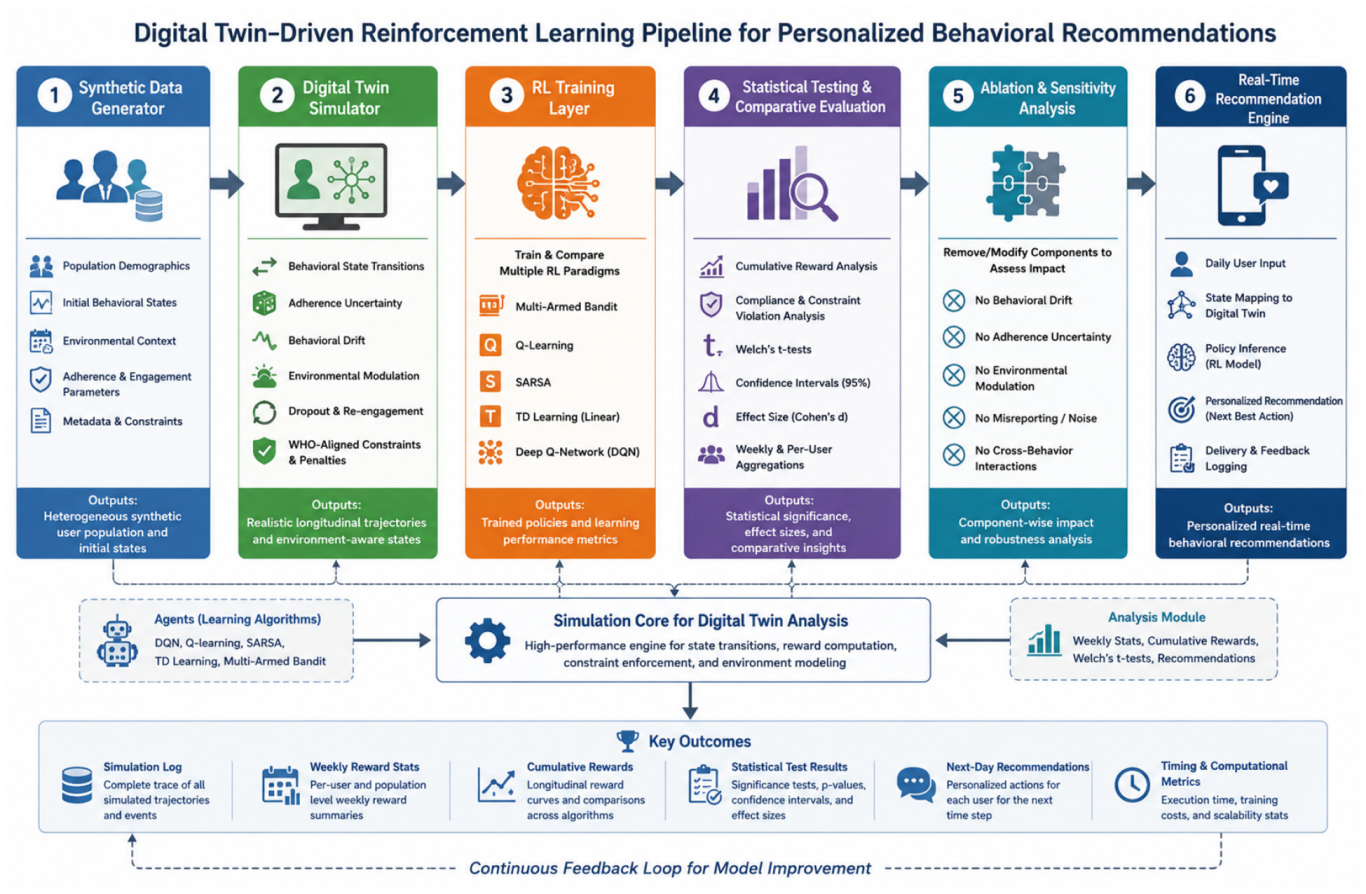
Pipeline architecture of the proposed digital twin-driven reinforcement learning framework for personalized behavioral recommendation generation.

The proposed framework follows a modular pipeline architecture consisting of six interconnected components: synthetic behavioral data generation, digital twin simulation, reinforcement learning training, comparative statistical evaluation, ablation-based sensitivity analysis, and real-time recommendation generation (see Figure 1). The synthetic data generation layer creates heterogeneous longitudinal user populations containing demographic attributes, behavioral states, environmental exposures, and engagement dynamics such as adherence uncertainty and dropout probability. These generated trajectories initialize the digital twin simulator, which models stochastic behavioral evolution, environmental modulation, behavioral drift, and WHO-aligned constraint dynamics over time. The reinforcement learning layer evaluates multiple learning paradigms under identical simulation conditions, including multi-armed bandits, Q-learning, SARSA, TD learning with function approximation, and DQN. The resulting policies are compared using cumulative effective reward, constraint violations, stability metrics, and computational cost. Statistical testing and effect-size analysis are subsequently performed to assess whether observed differences between methods are practically meaningful under stochastic behavioral variability. To further investigate the relationship between simulation realism and learning performance, the framework incorporates a structured ablation analysis module that selectively removes adherence uncertainty, behavioral drift, environmental modulation, and cross-behavior interactions. Finally, the trained reinforcement learning policies can be deployed through a lightweight recommendation engine capable of generating personalized real-time behavioral interventions from daily user inputs.

To contextualize the contributions of our digital twin framework, this study presents a qualitative comparison with existing methods in Table 1. To position this work within the existing literature, Table 1 provides a qualitative comparison between the proposed framework and representative categories of prior work. Unlike existing approaches that focus on either optimization or simulation in isolation, the present study integrates a behaviorally grounded digital twin with multiple reinforcement learning paradigms under a unified evaluation protocol.

**Table 1:** Qualitative comparison of the proposed study with related approaches in behavioral recommendation and reinforcement learning.

Approach category	Representative references	State representation	Action modeling	Dynamics and uncertainty	Evaluation rigor	Improvement in this study
Contextual/ multi-armed bandits	Tewari and Murphy, 2017; Liao et al., 2020	Shallow, mostly static context	Immediate, myopic actions	Typically stationary; limited adherence modeling	Short-horizon or single-metric	Bandits embedded as baselines within longhorizon digital twin simulations
Tabular RL for behavioral change	Sutton et al., 1998; Nahum-Shani et al., 2016	Discrete, coarse compliance states	Deterministic or weakly stochastic	Often assumes stationary transitions	Limited multi-run comparison	Explicit testing of tabular RL limits under richer dynamics
Deep RL for personalization	Raghu et al., 2017; Yu et al., 2021	Highdimensional continuous inputs	Abstract or black-box actions	Simulator realism often unclear	Offline or single-seed evaluation	Deep RL grounded in deficit-aware, interpretable digital twin dynamics
Agentbased/ simulationonly models	Bonabeau, 2002; Epstein, 2009	Rich agent states without optimization	Rule-driven interventions	Stochastic but non-adaptive	Descriptive analysis only	Simulation directly coupled with learning and statistical evaluation
Digital twin + RL (emerging)	Boschert and Rosen, 2016; Corral-Acero et al., 2020	Domainspecific twin models	Varies by application	Limited engagement modeling	Case-study driven	Unified experimental testbed enabling systematic RL comparison
This study	Proposed	Discrete + continuous behavioral, environmental, demographic state	Deficit- and context-aware actions	Adherence uncertainty, drift, dropout, re-engagement	Multi-seed, multi-user, statistical testing, ablation	Demonstrates when and why richer RL models outperform simpler baselines

The proposed framework uniquely integrates stochastic behavioral simulation, digital twin fidelity enhancements, context-aware reinforcement learning, and comparative statistical evaluation, which remain underexplored in prior research. Table 1 denotes a multi-dimensional continuous state vector comprising behavioral compliance indicators, normalized behavioral measures, environmental context variables, and demographic attributes. As shown in Table 1, unlike existing approaches that focus on either optimization or simulation in isolation, the present study integrates a behaviorally grounded digital twin with multiple reinforcement learning paradigms under a unified evaluation protocol. This work addresses these limitations through a unified architecture that combines digital twin simulation, stochastic behavioral modeling, reward optimization under uncertainty, and comparative reinforcement learning evaluation. This makes the proposed framework a scalable, testable, and extensible solution for studying adaptive behavioral recommendation strategies under realistic simulated conditions. This simulated approach not only accelerates development but also builds trust and transparency for future integration into clinical or health systems. The main study contributions are summarized as follows:

- We develop a digital twin-driven reinforcement learning framework for personalized behavioral recommendation generation under stochastic behavioral and environmental dynamics.
- We formulate personalized behavioral recommendation as a stochastic sequential decision problem within a Markov decision process (MDP) framework and comparatively evaluate multiple reinforcement learning paradigms under identical digital twin conditions.
- We introduce a simulation engine with user input validation, feedback generation, reward optimization, and accuracy adjustment capabilities to compensate for the limitations of static or short-term behavior models.
- Synthetic behavioral data modeled with adherence noise and dropout probabilities.
- All the simulations and respective code-bases will be publicly accessible for open-science research and improvements. The adopted simulation strategy can be used for synthetic behavioral data generation where data scarcity is a major concern!

The remainder of this paper is organized as follows: Section 2 details related work with novelty, Section 3 details the proposed work with problem formulation, Section 4 outlines adopted methodology, digital twin framework and its components, study simulation plan, and system description, Section 5 depicts experimental quantitative results and real-time application and Section 6 discusses experimental results with limitations, and future scope. The paper is concluded in Section 7.

## 2 Related work

This section explains the evolution of reinforcement learning methods from myopic approaches focused on short-term interventions to more expressive methods capable of simulating long-term behavioral dynamics. Early methods for context-aware, multi-stage slot machines demonstrated the feasibility of personalization under uncertainty, while tabular reinforcement learning introduced sequential thinking, but at the expense of relatively simple state abstraction. In recent years, although deep reinforcement learning methods have expanded their representational capabilities, they often rely on simplified simulators or offline data, limiting their interpretability and comparability. The parallel development of digital twins and simulation-based modeling techniques emphasizes realism and safety but often neglects systematic policy learning and comparative reinforcement learning evaluation.

### 2.1 Reinforcement learning for behavioral interventions

Reinforcement learning is increasingly being studied in the context of behavioral interventions and healthcare due to its ability to model sequential decision-making under uncertainty. Early work relied primarily on context-aware formulations and multi-armed bandits, particularly in mobile healthcare applications, where interventions are selected based on short-term feedback signals (Tewari and Murphy, 2017; Liao et al., 2020; Yu et al., 2021; Feng, 2024; Hu et al., 2023; Ganatra, 2025). Although these approaches are computationally efficient and suitable for online implementation, they largely ignore delayed and cumulative behavioral outcomes. To address these limitations, more expressive formulations based on Markov decision processes have been proposed. Reinforcement learning methods, including Q-learning and policy iteration, have been applied to coarse state abstractions based on self-assessment compliance or categorical health indicators (Sutton and Barto, 1998; Nahum-Shani et al., 2016). These studies demonstrate the feasibility of sequence personalization, but face scalability and information loss problems when behavioral states are over-discretized. Recent studies have explored deep reinforcement learning to leverage continuous sensor data streams and high-dimensional contextual data. For example, deep reinforcement learning (Q-learning) and Actor-Critic methods have been applied to personalized treatment strategies and adaptive interventions (Raghu et al., 2017; Yu et al., 2021). However, many of these studies rely on offline datasets or simplified simulators, making it difficult to assess their robustness, interpretability, and advantages over simpler baseline data.

### 2.2 Digital twins in human-centric systems

Digital twin technology has been extensively studied in engineering fields such as manufacturing and energy systems, where the laws of physics provide a solid foundation for simulation fidelity (Boschert and Rosen, 2016). However, in human-centered applications, digital twin technology faces additional challenges stemming from behavioral variability, partial observability, and feedback loops between system recommendations and user engagement. In healthcare, digital twin technology is primarily used for physiological modeling and disease progression analysis (Corral-Acero et al., 2020). These studies typically focus on short-term predictions or personalized treatment parameters rather than long-term decisions. Agent-based simulation methods have also been used to study population-level behavioral strategies and interventions (Bonabeau, 2002; Epstein, 2009; Katsoulakis et al., 2024; Łukaniszyn et al., 2024; Papachristou et al., 2024; Vall'ee, 2024); however, they often lack explicit policy optimization or learning components.

### 2.3 Simulation-based evaluation of reinforcement learning

Simulation environments are the cornerstone of reinforcement learning research, especially in robotics and control, enabling safe and repeatable experiments (Sutton and Barto, 1998). However, many test simulators are designed to improve algorithm performance rather than address domain-specific constraints, such as uncertainty in rule adherence, dynamic interactions, or trade-offs between multiple behaviors. In applied behavioral domains, this gap can lead to misleading conclusions about algorithmic superiority. As emphasized in recent methodological discussions, the evaluation environment must be sufficiently rich to justify the use of high-capacity models (Raghu et al., 2017). This research addresses this issue by designing a simulation platform focused on behavioral realism rather than algorithmic convenience.

### 2.4 Study contribution

This study contributes through the integration of digital twin simulation, statistically grounded behavioral modeling, and comparative reinforcement learning evaluation within a unified experimental framework. The novelty lies in combining realistic behavioral dynamics, engagement uncertainty, environmental modulation, and multi-paradigm reinforcement learning under controlled simulation conditions, thereby enabling systematic investigation of when higher-capacity learning methods provide meaningful benefits over simpler baselines. As shown in Table 1, the novelty of this work lies not in the development of new reinforcement learning algorithms, but in the systematic integration and evaluation of multiple learning paradigms in behavior-based digital twin models. Compared to previous work presented in Table 1, this study demonstrates that more refined state representations and more realistic action dynamics are necessary conditions for more advanced reinforcement learning methods to demonstrate significant advantages over simpler comparative methods.

## 3 Problem formulation

This study models personalized behavioral recommendations as a sequential decision problem within a simulated digital twin environment. Unlike many previous approaches (which either (i) independently optimize single behaviors or (ii) apply short-term heuristics while neglecting long-term planning), this model explicitly considers multi-criteria lifestyle management under uncertainty, including adherence differences, environmental changes, behavioral drift, and participation dynamics (disengagement and re-engagement). The goal is to develop a recommendation strategy that maximizes long-term health utility while minimizing violations of the behavioral and environmental constraints set by the World Health Organization (WHO).

### 3.1 Hypothesis statement

To support statistically grounded comparison among reinforcement learning paradigms, the study evaluates whether meaningful differences exist in cumulative effective reward across learning methods under identical digital twin conditions.

**Null hypothesis (*H*_0_):** there is no statistically significant difference in mean cumulative effective reward among the evaluated learning paradigms.


$$\begin{array}{l} H_{0}:\mu_{\text{Bandit}}=\mu_{\text{Q}}=\mu_{\text{SARSA}}=\mu_{\text{TD}}=\mu_{\text{DQN}} \end{array}$$

**Alternative hypothesis (*H*_1_):** at least one reinforcement learning paradigm achieves a statistically different mean cumulative effective reward under the simulated digital twin environment.


$$\begin{array}{l} H_{\text{1}}:\exists i,j\;\text{such}\;\text{that }\mu_{i}\neq \mu_{j} \end{array}$$

Pairwise *post-hoc* comparisons using Welch's *t*-tests, confidence intervals, and standardized effect sizes are additionally reported to evaluate practical differences between methods.

#### 3.1.1 State space

Depending on the learning algorithm, two complementary state encodings are used. The *discrete compliance state* is a binary vector indicating whether each of the eight behavioral goals consistent with the WHO guidelines is met. This gives 2^8^ = 256 possible states and supports tabular Q learning and SARSA. In parallel, a rich continuous state vector *x*_*t*_ ε R^*d*^ (where *d* = 26) is defined that combines: compliance bits, normalized continuous behavioral measures, normalized environmental variables, and demographic/contextual features. This richer representation enables function approximation TD learning and DQN to exploit continuous variation beyond binary compliance.

#### 3.1.2 Action space

The daily recommendation action set has been defined as:


$$\begin{array}{l} \text{A}=\{\text{sleep\_early},\text{ walk\_}30,\text{ eat\_veg\_}400,\text{ no\_tobacco\_alcohol},\\ \text{ meditate\_}10,\text{ drink water\_}8,\text{ digital\_detox\_}30,\text{ social\_}15\} \end{array}$$ (1)

#### 3.1.3 Transitions and uncertainty

The transition model *P* is induced by the digital twin and is explicitly stochastic. Action effects scale with the user's current deficits (e.g., larger improvements when far from the target), are modulated by environmental context (e.g., walking effectiveness reduced under poor air quality or hazards), and are gated by user adherence probability (e.g., non-adherence attenuates intended effects and may increase stress). Independent of actions, daily drift models habit decay and exogenous variability, and an engagement process models dropout and re-alignment.

### 3.2 Reward, penalty, and constraints

#### 3.2.1 WHO-aligned behavioral constraints

Let the primary behavioral targets be (Reuter et al., 2025; Mobasseri et al., 2025; Jayasinghe et al., 2025):


$$\begin{array}{l} \text{7}\leq \text{slee}{\text{p}}_{t}\leq \text{9},\;\text{activit}{\text{y}}_{t}\geq \text{3}0,\;\text{die}{\text{t}}_{t}\geq \text{4}00,\\ \text{abstai}{\text{n}}_{t}=\text{1},\;\text{wate}{\text{r}}_{t}\geq \text{8},\;\text{stres}{\text{s}}_{t}\leq \text{5},\\ \;\text{scree}{\text{n}}_{t}\leq \text{2},\;\text{socia}{\text{l}}_{t}\geq \text{15}. \end{array}$$ (2)

Additionally, simplified environmental constraints such as air quality, noise, night lighting and hazard indicators (e.g., heatwave/flood flags) are included, which influence the level of penalties and their effectiveness.

#### 3.2.2 Effective reward

At each day *t*, the health reward *r*_*t*_ is computed as a weighted function of compliance indicators and continuous stress quality. Constraint violations are penalized by a weighted indicator loss, producing an effective reward:


$$\begin{array}{l} \overset{\text{eff}}{\underset{t}{r}}=r_{t}-L_{t}, \end{array}$$ (3)

Where


$$\begin{array}{l} L_{t}=\;\overset{\left|C\right|}{\underset{j=1}{\sum }}\lambda_{j}\cdot 1\left[C_{j}\left(s_{t},a_{t}\right)\;\text{violated}\right] \end{array}$$ (4)

Here, *C*_*j*_ are WHO-aligned behavioral and environmental constraint functions, λ_*j*_ are penalty weights reflecting relative importance (e.g., higher penalties for harmful habits), and **1**[·] is the indicator function.

#### 3.2.3 Optimization objective

For a horizon of *T* days, the objective is to maximize the expected discounted cumulative effective reward:


$$\begin{array}{l} \underset{\pi }{\text{max}}{\text{E}}_{\pi }\left[\overset{T-\text{1}}{\underset{t=0}{\sum }}\gamma^{t}r_{t}^{\text{eff}}\right] \end{array}$$ (5)

where π(*a*|*s*) is the recommendation policy. In this experiment, *T* corresponds to the simulated intervention program duration (e.g., *T* = 365 days), and evaluation is performed using cumulative effective reward aggregated per user.

### 3.3 Learning methods and bellman optimality

All learning methods considered in this study are based on Bellman's optimality principle (Giuseppi and Pietrabissa, 2022; Mizutani and Dreyfus, 2023), which states that an optimal policy leads to optimal decisions in each successive state. Tabular Q-learning directly approximates the optimal state-action value function by iteratively applying the Bellman optimality operator, enabling greedy action selection based on expected long-term returns. SARSA relies on a similar temporal difference structure, but it evaluates actual policy actions, thus producing more conservative updates under stochastic transitions. TD learning, based on function approximation, extends the Bellman update to a continuous state space by learning a parameterized value function, while deep Q-learning generalizes this approach by using a nonlinear function approximator to capture complex state-action dependencies.

#### 3.3.1 Tabular Q-learning

For discrete states *s*_*t*_ ε {0,...,255} and actions *a*_*t*_ ε *A*, Q-learning updates the state-action value function using the Bellman optimality target:


$$\begin{array}{l} Q\left(s_{t},a_{t}\right)\leftarrow Q\left(s_{t},a_{t}\right)+\alpha \left[r_{t}^{\text{eff}}+\gamma \underset{a^{\prime }}{\text{max}}Q\left({s_{t}}_{+\text{1}},a^{\prime }\right)-Q\left(s_{t},a_{t}\right)\right], \end{array}$$ (6)

Where α is the learning rate. Under standard assumptions (e.g., sufficient exploration, appropriate step-size conditions, and stationary dynamics), Q-learning converges to the optimal action-value function. In this setting, stationarity is relaxed because the twin includes drift and engagement processes; thus, convergence has been interpreted as learning a robust near-optimal policy for the simulated environment, consistent with empirical RL practice.

#### 3.3.2 SARSA

SARSA uses an on-policy target:


$$\begin{array}{l} Q\left(s_{t},\;a_{t}\right)\leftarrow Q\left(s_{t},\;a_{t}\right)\;+\alpha \left[r_{t}^{\text{eff}}+\gamma Q\left({s_{t}}_{+\text{1}},\;{a_{t}}_{+\text{1}}\right)-Q\left(s_{t},\;a_{t}\right)\right], \end{array}$$ (7)

Which can yield more conservative behavior under stochastic transitions.

#### 3.3.3 TD learning with function approximation

For continuous state vectors *x*_*t*_ ε R^*d*^, TD(0) learns a value approximation *V*(*x*) = *w*^⊤^*x* via:


$$\begin{array}{l} w\leftarrow w+\alpha \left[r_{t}^{\text{eff}}+\gamma V\left({x_{t}}_{+\text{1}}\right)-V\left(x_{t}\right)x_{t}\right]. \end{array}$$ (8)

Action selection can be implemented via one-step lookahead by simulating approximate next-state effects under each candidate action.

#### 3.3.4 Deep Q-learning

Deep Q-learning approximates *Q*(*x, a*) using a neural network and trains with experience replay and a target network:


$$\begin{array}{lll} y & = & r_{t}^{\text{eff}}+\gamma \underset{a^{\prime }}{\text{max}}Q_{\theta }-\left({x_{t}}_{+\text{1}},a^{\prime }\right), \end{array}$$

$$\begin{array}{lll} \theta & \leftarrow & \text{arg}\underset{\theta }{\text{min}}𝔼\left[{\left(Q_{\theta }\left(x_{t},a_{t}\right)-y\right)}^{\text{2}}\right] \end{array}$$ (9)

#### 3.3.5 Multi-armed bandit baseline

As a non-sequential baseline, the multi-armed bandit learns a state-independent mean reward per action and selects actions via ϵ-greedy exploration. It provides a strong comparator when personalization is weak or when state information adds little value.

Let *A* = {*a*_1_, *a*_2_*,..., a*_*K*_} denote the set of *K* possible behavioral interventions (arms). At each day *t*, the learner selects an action *a*_*t*_ ε *A* and observes an effective reward:


$$\begin{array}{l} r_{t}^{\text{eff}}=r_{t}-L_{t}, \end{array}$$ (10)

Where *r*_*t*_ is the instantaneous health reward and *L*_*t*_ is the constraint-violation penalty defined in Equation 4. The reward distribution associated with each arm *a*_*k*_ is assumed to be stationary with unknown mean:


$$\begin{array}{l} \mu_{k}=𝔼\left[r_{t}^{\text{eff}}|a_{t}=a_{k}\right]. \end{array}$$ (11)

The objective of the bandit learner is to maximize the expected cumulative effective reward over a horizon of *T* days:


$$\begin{array}{l} \underset{{\left(a\right)}_{t=1}^{T}}{\text{max}}𝔼\left[\overset{T}{\underset{t=1}{\sum }}r_{t}^{\text{eff}}\right] \end{array}$$ (12)

In this study, we employ an ϵ-greedy strategy for action selection. At each day *t*, the action is chosen as:


$$\begin{array}{l} a=\;\{\begin{array}{cc} \text{uniformly}\;\text{random}\;\text{from }A\text{,} & \text{with}\;\text{probability}\;\epsilon ,\\ \text{arg}\;\text{ma}{{\text{x}}_{a_{k}}}_{\in A}{\hat{\mu }}_{k}\left(t\right), & \text{with}\;\text{probability}\;\text{1}-\epsilon , \end{array} \end{array}$$ (13)

Where μËĘ_*k*_(*t*) is the empirical mean effective reward of arm *a*_*k*_ up to time *t*:


$$\begin{array}{l} {\hat{\mu }}_{k}\left(t\right)=\frac{1}{N_{k}\left(t\right)}\overset{t}{\underset{\tau =1}{\sum }}r_{\tau }^{\text{eff}}\cdot 1\left[a_{\tau }=a_{k}\right], \end{array}$$ (14)

And *N*_*k*_(*t*) denotes the number of times arm *a*_*k*_ has been selected up to time *t*.

Unlike reinforcement learning models based on MDPs, multi-armed bandit models ignore state, delay effects, and state transition dynamics. Therefore, they cannot tailor recommendations to individual behavioral contexts or consider long-term trade-offs. Nevertheless, they still provide an important benchmark for evaluating whether increasing model complexity and state awareness significantly improves cumulative health outcomes in digital twin environments.

Although some previous studies have drawn conceptual analogies between behavioral scheduling and combinatorial routing problems such as the Traveling Salesman Problem (TSP), the present work does not formulate the recommendation task as a routing or permutation optimization problem. Instead, the proposed framework models behavioral recommendation as a stochastic sequential decision problem within a Markov Decision Process (MDP) environment, where state transitions depend on adherence uncertainty, environmental modulation, behavioral drift, and engagement dynamics. Furthermore, the daily behavioral actions in this model are not required to form Hamiltonian routes (there is no requirement that each behavior be visited exactly once), and the optimal strategy does not need to be a permutation. Therefore, in this study, the problem has not been transformed into a TSP and instead use RL to optimize expected long-term outcomes in realistic stochastic dynamics.

### 3.4 Proposed algorithm: stochastic reinforcement learning framework for digital twin behavioral recommendation

The Algorithm 1 formalizes constraint-aware learning (CARL) procedure for behavioral recommendations based on digital twins. This method goes beyond tabular Q-learning, supporting both discrete and continuous state representations, as well as multiple learning backends (tabular Q-learning/SARSA, TD with function approximation, and deep Q-learning). The main idea is to calculate an efficient reward by subtracting weighted penalties for violations of behavioral and environmental constraints, consistent with WHO guidelines. This generates a unified learning signal that promotes long-term health improvement while discouraging unsafe or undesirable behaviors. The algorithm runs in stages over a period of *T* days for each user, but can also be run online. At each time step, the policy uses a greedy ϵ approach to select an action, executes that action in the digital twin, observes the next state and its associated reward, evaluates the constraints, and updates the learner using the appropriate reinforcement learning update rules. The constraint module is considered a first-class component of the environment interface and can be extended with additional constraints without changing the learning logic.

Algorithm 1 CARL: constraint-aware reinforcement learning for digital twin behavioral recommendation. 
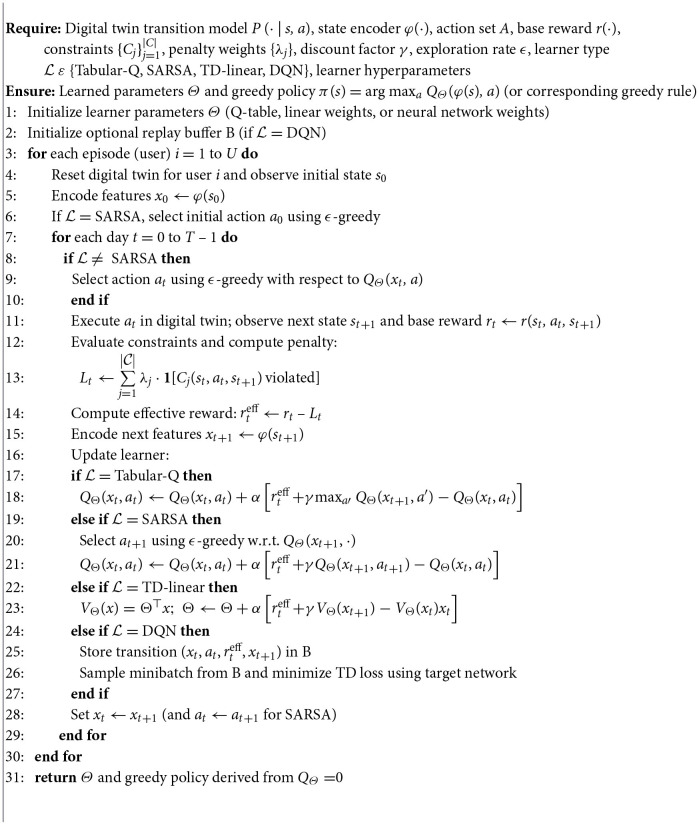

### 3.5 Computational complexity analysis

The computational complexity of CARL has been analyzed in terms of users *U*, horizon *T*, action count |*A*|, constraint count |*C*|, feature dimension *d*, replay buffer size *B*, and DQN minibatch size *m*. The total cost decomposes into (i) action selection, (ii) digital twin simulation, (iii) constraint evaluation, and (iv) learner updates.

#### 3.5.1 Common components

At each step, constraint evaluation requires checking |*C*| constraint functions, giving O(|*C*|) time. Digital twin simulation is a constant-time state update under bounded features, denoted O(1) per step [or O(*d*) if implemented as dense feature transforms]. Thus, the per-step baseline overhead is:


$$\begin{array}{l} O\left(|C|+d\right) \end{array}$$ (15)

#### 3.5.2 Action selection

Under ϵ-greedy, exploitation requires computing arg max_aε*A*_ *Q*_Θ_(*x, a*). For tabular Q and SARSA, this is O(|*A*|). For TD-linear with one-step lookahead over actions, if we evaluate all actions with simulated next-state features and a value function *V* (*x*), the cost becomes O(|*A*|·*d*) (each evaluation requires feature computation and a dot product). For DQN, greedy selection requires a forward pass producing all |*A*| *Q*-values; its cost is O(*F*) where *F* is the network forward complexity [e.g., *F* = O(*dh* + *h*^2^ + *h*|*A*|) for a two-hidden-layer MLP with width *h*].

#### 3.5.3 Learner update costs

- **Tabular-Q/SARSA:** update requires a max over actions (Q-learning) or one next-action lookup (SARSA), giving O(|*A*|) or O(1), respectively for the target, and O(1) for the update assignment. Overall per-step update is O(|*A*|) for Q-learning and O(1) for SARSA target computation once *a*__*t*_+1_ is chosen.
- **TD-linear:** update is a dot product and weight update, O(*d*) per step.
- **DQN:** storing transitions is O(1); training requires sampling minibatches and backpropagation. Per update step is O(*m*·(*F* + *Bwd*)), where *Bwd* is the backpropagation cost (on the same order as *F*). Thus, DQN update complexity is approximately O(*m*·*F*) per gradient step.

#### 3.5.4 Total runtime

Let κ_sim_ denote the constant (or O(*d*)) digital twin update cost. Then the total runtime over all users and days is:

Tabular Q-learning


$$\begin{array}{l} O(UT(|C|+\kappa_{\text{sim}}+|A|)), \end{array}$$ (16)

where the |A| term arises from computing $\underset{a^{\prime }\in A}{\max}Q\left(s_{t+1},a^{\prime }\right)$ in the TD target.

SARSA


$$\begin{array}{l} O(UT(|C|+\kappa_{\text{sim}})), \end{array}$$ (17)

assuming the next action *a*_*t*+1_ is already selected by the behavior policy. The SARSA TD target, *Q*(*s*_*t*_, *a*_*t*_) ← *Q*(*s*_*t*_, *a*_*t*_) + α[*r*_*t*_ + γ*Q*(*s*_*t*+1_, *a*_*t*+1_) − *Q*(*s*_*t*_, *a*_*t*_)], is then O(1).

TD-linear (no lookahead)


$$\begin{array}{l} O\left(UT\left(|C|+\kappa_{\text{sim}}+d\right)\right). \end{array}$$ (18)

TD-linear with one-step lookahead


$$\begin{array}{l} O\left(UT\left(|C|+\kappa_{\text{sim}}+|A|d\right)\right) \end{array}$$ (19)

DQN (with minibatch updates each step)


$$\begin{array}{l} O\left(UT\left(|C|+\kappa_{\text{sim}}+F+mF\right)\right)\approx O\left(UT\left(|C|+\kappa_{\text{sim}}+mF\right)\right). \end{array}$$ (20)

#### 3.5.5 Space complexity

Tabular methods require O(|*S*||*A*|) storage for the Q-table (with |*S*| = 256 for compliance states). TD-linear requires O(*d*) memory for weights. DQN requires storing network parameters O(*P*) (with *P* weights) and a replay buffer of size O(*B*·(*d* + 1)) for storing transitions.

#### 3.5.6 Practical implications

The analysis highlights that tabular and bandit methods are computationally efficient and scale well to large populations. TD-linear is attractive when *d* is moderate and lookahead is avoided. DQN provides the highest representational capacity but has a significantly higher update cost due to training in mini-batches, limiting its use for large-scale updates. This motivates the reporting of both performance metrics (cumulative effective reward) and system metrics (latency and execution time) when comparing algorithms in digital twin pipelines.

## 4 Methodology

This section presents the complete methodology used to design, implement, and evaluate the proposed digital twin-based reinforcement learning framework for personalized behavioral recommendations. The methodology emphasizes realism, repeatability, and statistical rigor by combining rule-based synthetic data generation, constraint-aware simulation, multiple reinforcement learning paradigms, and systematic experimental evaluation. Unlike real-world observational studies, the use of a digital twin enables controlled experimentation under strict assumptions while preserving significant behavioral complexity. The methodological process consists of six steps: (i) rule-based synthetic dataset generation, (ii) digital twin construction and initialization, (iii) richer state and action-effect modeling, (iv) reinforcement learning and bandit optimization, (v) hyperparameter selection via grid search, and (vi) experimental evaluation with statistical testing. Each stage is modular, allowing for independent modification and ablation without changing the overall structure. The methodology provides a controlled yet behaviorally rich digital twin simulation environment and a unified evaluation framework for comparing multiple reinforcement learning paradigms. Combining the effects of deficit-based interventions, adherence and engagement uncertainty, drift, misreporting, multilevel assessment, and ablation allows for rigorous examination of when more complex RL models yield measurable benefits compared to simpler benchmarks in personalized behavioral recommendations.

### 4.1 Pipeline architecture of the proposed framework

The overall framework is organized as a modular pipeline architecture designed to separate data generation, simulation, learning, evaluation, and deployment stages. This modular organization improves reproducibility, interpretability, and extensibility while enabling controlled experimental analysis of reinforcement learning behavior under realistic digital twin dynamics. Figure 1 presents the complete pipeline architecture of the proposed framework, illustrating how synthetic data generation, digital twin simulation, reinforcement learning optimization, statistical evaluation, ablation analysis, and real-time recommendation generation are integrated into a unified experimental and deployment workflow.

#### 4.1.1 Stage 1: synthetic data generator

The first stage generates heterogeneous synthetic user populations with demographic variables, behavioral characteristics, environmental exposures, and engagement parameters. Longitudinal trajectories are initialized using constrained stochastic processes to ensure behavioral realism and diversity across users.

#### 4.1.2 Stage 2: digital twin simulator

The digital twin simulation engine models daily behavioral evolution using stochastic transitions influenced by adherence uncertainty, behavioral drift, environmental context, dropout, and re-engagement dynamics. WHO-aligned behavioral constraints and environmental penalties are integrated directly into the state-transition process.

#### 4.1.3 Stage 3: reinforcement learning training layer

The reinforcement learning layer trains and compares multiple learning paradigms under identical simulation conditions. The evaluated methods include multi-armed bandits, tabular Q-learning, SARSA, TD learning with linear approximation, and Deep Q-Networks. The learning objective is defined through cumulative effective reward optimization under constraint-aware behavioral dynamics.

#### 4.1.4 Stage 4: statistical testing and comparative evaluation

The evaluation stage aggregates cumulative rewards, compliance trajectories, and constraint violations across users and random seeds. Statistical analysis includes Welch's *t*-tests, confidence intervals, and effect-size estimation to assess practical differences between algorithms under stochastic behavioral variability.

#### 4.1.5 Stage 5: ablation and sensitivity analysis

The ablation module evaluates the contribution of individual simulation components by selectively removing behavioral drift, adherence stochasticity, environmental modulation, and cross-behavior interactions. This stage assesses whether performance gains reflect genuine learning advantages or simplified environmental difficulty.

#### 4.1.6 Stage 6: real-time recommendation engine

The final stage demonstrates translational feasibility through deployment of trained reinforcement learning policies within a lightweight recommendation interface. Daily behavioral inputs are mapped into digital twin states, and the trained policy generates context-aware personalized recommendations in real time.

### 4.2 Dataset description and generation

This sub-section describes the nature of the simulated datasets with demography, generation policy, and temporal structure.

#### 4.2.1 Synthetic population dataset

A synthetic dataset representing a heterogeneous user population is generated. Each synthetic individual is characterized by demographic attributes (e.g., age, gender, body mass index), basic lifestyle behaviors (e.g., sleep, activity, diet, hydration, stress), contextual variables (e.g., work schedule, screen exposure), and engagement parameters (e.g., likelihood of adherence, likelihood of dropping out, likelihood of re-engagement). The dataset is generated using deterministic rules combined with stochastic perturbations to ensure diversity while maintaining plausibility. Tables 2–9 elaborate the demographic attributes, state variables, actions, rewards, penalties, and derived monitoring signals used throughout the digital twin simulation and reinforcement learning evaluation. Furthermore, Table 10 provides a summarization of generated dataset features with behavioral variables.

**Table 2:** Demographic and user profile variables used in the digital twin.

Variable	Type	Range/ values	Role in simulation
Age	Continuous	18–64 (years)	Shapes baseline sleep, activity, and stress dynamics
Sex	Categorical	Male/female	Stratification and baseline heterogeneity
BMI	Continuous	18.5–40 (kg/m^2^)	Influences activity capacity and reward scaling
Work schedule	Categorical	Standard/shift	Modulates sleep regularity and stress drift
Smoker status	Binary	0/1	Affects abstinence constraint and baseline behaviors

**Table 3:** Engagement and compliance parameters governing user response.

Parameter	Type	Description
Adherence propensity	Continuous (0, 1)	Probability that a recommended action is executed as intended
Weekly dropout probability	Continuous (0, 1)	Probability of temporary disengagement from the program
Weekly realignment probability	Continuous (0, 1)	Probability of re-engaging after dropout

**Table 4:** Behavioral state variables forming the digital twin internal state.

Variable	Type	Units/range	Use
Sleep duration	Continuous	Hours/night	Compliance, reward, penalty
Physical activity	Continuous	Minutes/day	Compliance, reward
Diet quality	Continuous	Grams fruits/veg	Compliance, reward
Abstinence	Binary	0/1	Habit constraint and reward
Stress level	Continuous	0–10	Continuous reward and drift
Mindfulness	Continuous	Minutes/day	Stress reduction dynamics
Hydration	Discrete	Glasses/day	Compliance and reward
Screen time	Continuous	Hours/day	Penalty above threshold
Social interaction	Continuous	Minutes/day	Lifestyle balance indicator

**Table 5:** Environmental context variables affecting penalties and action effects.

Variable	Type	Range	Effect
Air quality index	Continuous	≥ 0	Penalizes outdoor actions when high
Noise level	Continuous	dB	Affects sleep penalties
Night light exposure	Continuous	Lux	Affects sleep quality
Eco-anxiety	Continuous	0–10	Increases stress dynamics
Heatwave	Binary	0/1	Reduces action effectiveness
Flood	Binary	0/1	Limits feasible interventions

**Table 6:** Action space representing daily behavioral recommendations.

Action	Intended effect
sleep_early	Improves sleep duration and quality
walk_30	Increases physical activity, reduces stress
eat_veg_400	Improves diet quality
no_tobacco_alcohol	Enforces abstinence from harmful habits
meditate_10	Reduces stress and improves mental health
drink_water_8	Improves hydration
digital_detox_30	Reduces screen time
social_15	Increases social interaction

**Table 7:** Reward components and their interpretation.

Reward component	Description
Sleep reward	Granted when sleep duration is within target range
Activity reward	Granted when activity target is met
Diet reward	Granted when dietary intake meets threshold
Habit reward	Granted for abstinence from tobacco/alcohol
Stress reward	Continuous reward for lower stress levels
Hydration reward	Granted when hydration target is met
Screen reward	Granted for limiting screen time
Social reward	Granted for sufficient social interaction

**Table 8:** Penalty components corresponding to violated constraints.

Penalty	Triggered when
Sleep penalty	Sleep outside recommended range
Activity penalty	Insufficient daily activity
Diet penalty	Inadequate fruit/vegetable intake
Habit penalty	Tobacco or alcohol use
Hydration penalty	Insufficient water intake
Mental penalty	High stress or low mindfulness
Screen penalty	Excessive screen time
Social penalty	Insufficient social interaction
Environmental penalties	Unsafe air quality, noise, light, or hazards

**Table 9:** Derived variables used for monitoring and evaluation.

Variable	Purpose
Effective reward	Reward minus total penalties; primary optimization signal
Compliance flags	Binary indicators for WHO guideline adherence
Constraint violation count	Daily number of violated constraints
Weekly reward mean/std	Learning dynamics and stability analysis
Dropout status	Indicates engagement or disengagement
Behavioral deficits	Distance from targets; used for action-effect scaling

**Table 10:** Summary of dataset features and behavioral variables.

Category	Variables
Demographics	Age, sex, BMI
Behavioral states	Sleep, activity, diet, hydration, stress, screen time, social interaction
Habits	Tobacco/alcohol abstinence
Environment	Air quality, noise, night light, hazard indicators
Engagement	Adherence probability, dropout, realignment

#### 4.2.2 Rule-based generation with constraints

Behavioral variables are sampled from constrained distributions consistent with public health guidelines. Hard constraints ensure values fall within physiologically acceptable ranges (e.g., sleep hours from 4 to 12, stress scores from 0 to 10), while soft constraints correspond to targets consistent with WHO guidelines, which are then used to calculate rewards and penalties. Environmental variables (e.g., air quality, noise, light exposure, threat indicators) are generated independently but influence both penalties and the effectiveness of interventions.

#### 4.2.3 Temporal structure

The dataset is longitudinal. For each user, a daily trajectory is generated over the full simulation horizon (typically *T* = 365 days). Initial values are sampled at time *t* = 0, and subsequent values evolve based on action effects, stochastic policy compliance, and natural drift. This structure allows for the assessment of long-term learning and policy stability.

### 4.3 Statistical validation of generated data

To ensure that the synthetically generated data are statistically significant, internally consistent, and suitable for machine learning-based optimization, we conduct a comprehensive validation protocol before starting the machine learning run. This validation aims to exclude degenerate distributions, unrealistic independence assumptions, and spurious action effects that could bias the results. All tests are conducted using the generated population demographics, initial states, and (if available) the full behavioral trajectories.

#### 4.3.1 Descriptive statistics and boundedness checks

In the first step, descriptive statistics (e.g., mean, standard deviation, minimum, and maximum) are calculated for all demographic, behavioral, and environmental variables. This verification confirms that all generated values fall within predefined physiological and behavioral probability bounds. To detect potential saturation artifacts caused by truncation, we additionally calculate the percentage of observations that fall exactly within the minimum or maximum bounds for each variable. A low probability of hitting the bound indicates that the variables are not artificially constrained and retain sufficient variability for learning.

#### 4.3.2 Distributional goodness-of-fit tests

For continuous demographic variables such as age and BMI, we test whether their empirical distributions conform to the assumed generative assumptions. Specifically, we conduct Kolmogorov–Smirnov tests (Berger and Zhou, 2014) against fitted normal distributions and Shapiro–Wilk tests (Gonz'alez-Estrada and Cosmes, 2019) on subsamples to assess deviations from normality. For probabilistic engagement parameters (e.g., adherence propensity), we fit beta distributions (Johnson et al., 1994) and use Kolmogorov–Smirnov tests to verify whether sample values conform to the assumed constrained stochastic structure. These tests ensure that the synthetic population exhibits realistic dispersion and shape rather than arbitrary or pathological distributions.

#### 4.3.3 Proportion and constraint consistency tests

For binary or categorical variables (e.g., smoking status, abstinence rates), we calculate empirical proportions and their associated 95% binomial confidence intervals. This check verifies that the category frequencies are not degenerate or improbably skewed. Furthermore, we calculate mean behavioral deficits relative to WHO-guided targets (e.g., sleep, activity, diet) to confirm that the initial population includes both adherent and nonadherent individuals, justifying the need for personalized intervention strategies.

#### 4.3.4 Dependency and face-validity tests

To establish the visual validity of the relationships encoded in the generator, we conduct targeted tests of association. For example, we examine whether nonsmokers demonstrate higher baseline physical activity than smokers using Welch's *t-*test, and whether smoking status is statistically associated with abstinence rates using the chi-square test of independence. We then examine whether baseline stress parameters predict initial stress levels using linear regression. Statistically significant results in the expected directions confirm that the generator preserves plausible behavioral relationships rather than generating independent noise.

#### 4.3.5 Temporal dependence and drift

Temporal dependence is assessed by examining lagged correlations in behavioral trajectories (when full trajectories are available). The presence of positive autocorrelation in variables such as activity, sleep, and stress confirms habit persistence and gradual drift, distinguishing simulation from independent and identically distributed noise. This temporal structure is necessary for the learning problem to be truly sequential.

#### 4.3.6 Action-effect validation

To validate that simulated actions produce meaningful and context-dependent effects, we compare next-day behavioral changes under targeted interventions vs. control actions. For example, when activity deficit is high and environmental conditions are favorable, we test whether the walk 30 action leads to a significantly larger increase in activity minutes than non-walking actions. Welch's *t*-test is used to compare action-induced deltas under matched contextual conditions. Statistically significant differences confirm that actions have causal impact rather than acting as symbolic labels.

#### 4.3.7 Hypothesis testing on generated data

Formally, for action-effect validation we test:


$$\begin{array}{l} H_{0}:\mu_{\text{adhered}}=\mu_{\text{non-adhered}},H_{\text{1}}:\mu_{\text{adhered}}>\mu_{\text{non-adhered}}, \end{array}$$ (21)

Where μ_adhered_ and μ_non − adhered_ denote the mean effective reward (or behavioral delta) under adhered and non-adhered executions of the same intervention. Welch's *t*-test is employed to account for unequal variances and sample sizes. Rejection of *H*_0_ provides statistical evidence that the digital twin environment encodes meaningful intervention effects rather than random fluctuations.

#### 4.3.8 Variance decomposition and personalization justification

Finally, we decompose the variance of rewards and behavioral outcomes into between-user and within-user components. The presence of significant between-user variance confirms population heterogeneity and justifies the use of personalized, state-dependent policies instead of global heuristics. Collectively, these statistical validations demonstrate that the generated dataset is non-degenerate, behaviorally plausible, temporally structured, and suitable for rigorous evaluation of reinforcement learning-based recommendation strategies.

### 4.4 Digital twin construction

A population of synthetic users is generated, representing heterogeneous demographic and behavioral characteristics. Each user is defined based on demographic data (e.g., age, gender, BMI), behavioral predispositions (baseline activity, baseline stress), contextual factors (e.g., work schedule such as shift/non-shift work), and engagement parameters (e.g., likelihood of compliance, likelihood of skipping weekly activities, likelihood of reorganization). The digital twin's state is updated daily and includes multiple behavioral dimensions, including sleep duration, minutes of physical activity, fruit and vegetable consumption, tobacco/alcohol abstinence, perceived stress level (ε [0, 10]), mindfulness minutes, hydration, screen time, and minutes of social interaction. Variables related to environmental exposure (e.g., air quality, noise, nighttime lighting, ecological anxiety, and threat flags such as heatwave/flood) are also maintained to enable context-dependent effectiveness of interventions and the imposition of penalties for restrictions. Table 11 defines the components of the conceptualized digital twin framework.

**Table 11:** Components of the digital twin framework.

Component	Description
User model	Demographics (age, sex, BMI), context (work schedule), baseline behaviors, adherence propensity, dropout/realignment dynamics
Behavioral state	Sleep, activity, diet, habit (abstinence), stress, mindfulness, hydration, screen time, social interaction, plus environmental exposures
Action space	Eight WHO-aligned daily interventions (sleep hygiene, activity, diet, abstinence, mindfulness, hydration, screen reduction, social interaction)
Reward/penalty model	Compliance-based reward with continuous stress quality; penalties for behavioral and environmental constraint violations
Agents	Bandit (ϵ-greedy), Q-learning, SARSA, TD learning (linear approximation), deep Q-learning (DQN)
Evaluation protocol	Multi-seed simulation, user-level cumulative reward, weekly curves, constraint violations, dropout-adjusted compliance, statistical testing, ablation

#### 4.4.1 State initialization and feasible ranges

Initial states are sampled from constrained distributions and trimmed to plausible physiological and behavioral ranges to ensure numerical stability and avoid degenerate trajectories. These ranges are aligned with daily goals used to assess adherence (e.g., sleep, activity, diet, hydration) and are then used to normalize continuous features in function approximation models. This initialization creates a diverse cohort with diverse deficit patterns, enabling significant personalization.

#### 4.4.2 Engagement dynamics: adherence, dropout, and realignment

A key aspect of the twin model is the explicit modeling of engagement. Each recommended action is implemented with a probability equal to the user's propensity to adhere; otherwise, attenuated or adverse outcomes may occur (e.g., nonadherence, which increases stress). Furthermore, a weekly Bernoulli process of disengagement models temporary disengagement, during which users ignore the recommendations and actions are essentially random. The weekly reengagement process allows for re-engagement, reinstating adherence-based behavior. This mechanism induces patterns of non-stationarity and disengagement similar to those observed in long-term behavior change programs.

### 4.5 Richer state and action-effect modeling

A central design goal is to ensure that higher-capacity models (TD learning and DQN) can exploit additional structure unavailable to simpler baselines.

#### 4.5.1 State representation

Two complementary state representations are used. The discrete compliance state encodes binary indicators for WHO-compliant targets, creating a compact state space suitable for tabular RL. In parallel, a richer, continuous state vector (26 dimensions) combines compliance indicators, normalized behavioral measures, environmental context, and demographic characteristics. This representation enables generalization across users and contexts.

#### 4.5.2 Action-effect structure

Actions are deficit-aware and context-dependent. The magnitude of the effect scales with the current behavioral deficit and is modulated by environmental conditions. Interactions between behaviors introduce trade-offs (e.g., increased activity can reduce available sleep time), preventing trivial solutions and creating a truly multi-objective optimization problem.

#### 4.5.3 Importance of capacity

In this design, bandit and tabular methods can only respond to coarse vulnerability patterns, while TD and DQN methods can condition decisions based on subtle changes in stress, environment, and user context. This ensures that performance differences reflect the algorithm's capabilities rather than artifacts of an impoverished state space.

#### 4.5.4 Discrete compliance state and rich continuous state

We employ two state encodings. The discrete compliance state is an 8-bit vector of compliance flags consistent with WHO guidelines (e.g., sleep, activity, diet, habit, a proxy for mental health, hydration, screen time, and social interactions), resulting in a 256-state representation adapted to tabular RL. Furthermore, a continuous state vector of dimension *d* = 26 combines compliance flags, normalized continuous behavioral measures, normalized environmental variables, and normalized demographic/-context indicators. This dual representation allows for a controlled comparison of algorithms based on discrete aggregation with those using continuous context.

#### 4.5.5 Deficit-aware, context-dependent action effects

Each action affects multiple behavioral dimensions and is explicitly deficit-aware, meaning its magnitude depends on the user's proximity to the goal. For example, physical activity recommendations yield greater benefits when activity deficits are high; sleep hygiene recommendations have a greater impact when sleep is below the goal; and mindfulness-based actions reduce stress more effectively when stress levels are elevated. Environmental context modulates action effectiveness (e.g., outdoor walks yield less benefit under high air quality or extreme conditions). Trade-offs between behaviors are also modeled (e.g., dietary changes may reduce social minutes; limiting screen use may increase social interactions), preventing trivial dominance of a single action and creating a genuine multi-criteria control problem.

#### 4.5.6 Drift and misreporting

Regardless of the intervention, daily drift processes capture habit decay and exogenous variability (e.g., gradual increases in screen time, declines in physical activity, fluctuations in stress levels). Misreporting is modeled as additive noise in observed reward signals, reflecting inconsistencies in self-reported data and creating partial observability. This ensures that strategies must learn under conditions of perturbative feedback rather than exploiting deterministic dynamics.

### 4.6 Grid-search for hyperparameter selection

Hyperparameters are selected using a structured grid search, conducted entirely in a simulation environment. For each algorithm, candidate values for the learning rate, discount factor, and exploration factor are evaluated in short pilot runs using multiple random seeds. The selection criterion is the average cumulative effective reward with variance regularization, which avoids unstable configurations. To ensure methodological fairness and prevent overfitting to a specific agent, a single default configuration is adopted as the final setting for all algorithms. Default configuration from has been summarized in Table 12.

**Table 12:** Default hyperparameter configuration selected as best across all learning agents.

Algorithm	α	γ	ϵ	αTD	DQN learning rate	DQN hidden units
Q-learning	0.25	0.97	0.12	0.05	1 × 10^−3^	128
SARSA	0.25	0.97	0.12	0.05	1 × 10^−3^	128
TD learning	0.25	0.97	0.12	0.05	1 × 10^−3^	128
Deep Q-network (DQN)	0.25	0.97	0.12	0.05	1 × 10^−3^	128
Multi-armed bandit	0.25	0.97	0.12	0.05	1 × 10^−3^	128

A unified configuration has been adopted to ensure fair comparison and avoid algorithm-specific overfitting.

### 4.7 Reinforcement learning framework: agents and learning paradigms

The optimization problem is formulated as an MDP, where each day represents a decision step. Actions correspond to daily behavioral interventions, and the environment represents the dynamics of the digital twin. The goal is defined by the effective reward, calculated as the base reward minus penalties for constraint violations. This study methodology focuses on reinforcement learning and bandit baselines, adapted to the implemented simulation environment. Five learning paradigms are evaluated under identical conditions: (1) multi-armed bandit with ϵ-greedy exploration as a non-sequential baseline, (2) tabular Q-learning (off-policy), (3) SARSA (on-policy), (4) TD learning with linear function approximation for continuous states, and (5) deep Q-learning (DQN) for nonlinear value approximation. The use of both discrete and continuous state representations ensures that performance differences can be attributed to algorithmic capacity rather than differences in observability or logging. Table 13 defines the used algorithms compared in the conceptualized digital twin framework.

**Table 13:** Algorithms compared in the digital twin framework.

Algorithm	Type	Key characteristics
Bandit (ϵ-greedy)	Non-sequential baseline	Learns state-independent mean reward per action; cannot personalize to state
Q-learning	Off-policy RL	Tabular value learning on discrete compliance states; optimizes long-horizon effective reward
SARSA	On-policy RL	Conservative value learning; policy-coupled updates under ϵ-greedy exploration
TD (linear approx.)	Approximate RL	Learns *V*(*x*) on continuous state vector; supports generalization across users and contexts
Deep Q-learning (DQN)	Deep RL	Neural approximation of *Q*(*x, a*) using replay buffer and target network; captures non-linear state-action interactions

#### 4.7.1 DQN architecture and training procedure

The DQN implementation employs a fully connected multilayer perceptron with two hidden layers of 128 rectified linear units (ReLU) each. The input layer receives the 26-dimensional continuous state representation, and the output layer predicts Q-values for all behavioral actions. Experience replay is implemented using a replay buffer with uniform random sampling, and target-network synchronization is performed periodically every 100 update steps to stabilize training. Mini-batch gradient descent is performed using the Adam optimizer with learning rate 5 × 10^−4^. The replay batch size is set to 64, and ϵ-greedy exploration decays gradually during training.

#### 4.7.2 Effective reward and constraint penalties

Rewards are constructed to reflect WHO-aligned compliance and stress quality, while penalties quantify violations of behavioral and environmental thresholds. The effective reward is:


$$\begin{array}{l} \overset{eff}{\underset{t}{r}}=r_{t}-\sum_{j}\lambda_{j}1\;[C_{j}\text{ violated}], \end{array}$$ (22)

Where *C*_*j*_ are constraint functions (e.g., sleep range, minimum activity, diet threshold, abstinence, hydration, stress limit, screen cap, social minimum, and environmental thresholds) and λ_*j*_ are tunable penalty weights. This ensures the policy is optimized for sustainable multi-behavior improvement rather than maximizing a single behavioral dimension. Table 14 defines the utilized action, reward, and penalty structure.

**Table 14:** Action, reward, and penalty structure.

Component	Description
Actions	Eight WHO-aligned daily interventions
Reward	Compliance-based health utility + stress quality
Penalties	Weighted violations of behavioral and environmental constraints
Effective reward	Reward minus total penalty

### 4.8 Simulation planning and evaluation protocol

Each experiment simulates all users over a horizon of *T* = 365 days. Each day includes observing the state, selecting an agent action, applying action effects depending on compliance, updating the state via drift, calculating the reward/penalty, and logging. To reduce sensitivity to a single random trajectory, all results are aggregated across multiple independent random seeds (multi-pass evaluation), and uncertainty is reported using means and standard deviations across seeds and users.

#### 4.8.1 Logging, aggregation, and derived metrics

For each agent and user, daily logs include state variables, chosen action, base reward, penalty, effective reward, compliance rates, environmental compliance rates, and churn status. Aggregations are computed at multiple temporal resolutions, including weekly averages, to analyze learning dynamics and stability. The main output is the cumulative effective reward for each user over the entire time horizon, which is then compared across agents. Additional metrics include constraint violation frequency, churn-corrected compliance rate, and learning curve stability (weekly mean ± standard deviation across seeds).

#### 4.8.2 Statistical testing

To compare reinforcement learning paradigms, statistical analyses are conducted using per-user cumulative effective reward as the primary unit of analysis. Welch's *t*-tests are employed to account for unequal variances and sample sizes across learning methods. The statistical evaluation is designed to assess whether meaningful differences exist among the evaluated reinforcement learning paradigms under identical digital twin simulation conditions.

Formally, the comparative evaluation considers:


$$\begin{array}{l} H_{0}:\mu_{\text{Bandit}}=\mu_{\text{Q}}=\mu_{\text{SARSA}}=\mu_{\text{TD}}=\mu_{\text{DQN}}, \end{array}$$ (23)

against the alternative hypothesis that at least one learning paradigm exhibits statistically different cumulative effective reward:


$$\begin{array}{l} H_{1}:\exists_{i,j}\;\text{such that }\mu_{i}=\mu_{j}. \end{array}$$ (24)

To complement global hypothesis testing, pairwise *post-hoc* comparisons between DQN and alternative learning paradigms are additionally evaluated using Welch's *t*-tests, 95% confidence intervals, and Cohen's *d* effect-size estimates. These analyses provide both statistical and practical interpretation of observed reward differences under stochastic behavioral variability.

#### 4.8.3 Evaluation metrics

Performance is evaluated using cumulative effective reward as the primary metric. Secondary metrics include constraint violation frequency, compliance rate, weekly reward trends, and computational cost. Statistical comparisons between methods are performed using Welch's *t*-test at the user level. Table 15 defines the adopted metrics for performance evaluation.

**Table 15:** Adopted metrics for performance evaluation.

Metric	Description
Cumulative effective reward	Per-user sum of (reward–penalty) across *T* days; primary out-come for statistical testing
Constraint violations	Frequency of WHO-aligned behavioral and environmental constraint violations
Weekly reward curves	Weekly mean ± standard deviation across seeds; indicates learning stability and temporal dynamics
Dropout-adjusted compliance	Proportion of compliant days accounting for dropout/realignment status
Statistical tests	Welch's *t*-test (one-sided or two-sided), effect size estimates, confidence intervals
Computational cost	Cumulative action-selection and update time; supports system-level tradeoff analysis

### 4.9 Ablation study design

To identify which components of the digital twin environment are essential for the observed performance of DQN, we conduct a structured ablation study. Each ablation selectively removes or simplifies a single mechanism while keeping all other simulation parameters, hyperparameters, and random seeds fixed. This design isolates causal contributions and avoids confounding effects. Specifically, we evaluate the following ablations. *No Adherence Stochasticity* enforces perfect compliance with recommended actions, removing execution uncertainty. *No Behavioral Drift* eliminates gradual decay and long-term habit erosion, resulting in stationary state dynamics. *No Environmental Modulation* disables context-sensitive action effects, causing interventions to have identical impact regardless of environmental conditions. *No Cross-Behavior Effects* restricts actions to affect only a single behavioral dimension, removing trade-offs between behaviors such as sleep, activity, and stress. Finally, *No Misreporting Noise* removes observation noise, making perceived states identical to true states. By comparing these simplified settings against the full model, the ablation study evaluates whether DQN's advantages persist under reduced complexity and clarifies the conditions under which higher-capacity reinforcement learning models provide meaningful benefits over simpler baselines. This analysis also guards against attributing performance gains solely to model capacity rather than environment structure.

### 4.10 Data pipeline and implementation software

The pipeline is implemented primarily in Python, using NumPy for simulation and array-based updates, Pandas for tabular logging and aggregation, SciPy for hypothesis testing, and Matplotlib for visualization. Deep Q-learning can be implemented using TensorFlow/Keras for neural function approximation when GPU acceleration is available. For scalable execution, simulation across users and seeds can be parallelized using distributed compute frameworks such as Dask or Apache Spark (PySpark), and workflow orchestration can be managed with tools such as Airflow or Prefect. Results are stored in structured formats (CSV/JSON) and optionally in columnar formats for scale. Version control and reproducibility are maintained via Git, containerization via Docker, and experiment tracking can be supported through MLflow.

### 4.11 System interface and translational feasibility

To demonstrate translational feasibility, a trained reinforcement learning policy can be deployed via a lightweight web service that accepts daily user input and returns the highest-rated recommendations. The interface layer is intentionally modular and decoupled from the simulation engine, allowing for *in-silico* evaluation of the same policy and subsequent integration with systems. Although this paper focuses on simulation and optimization methodology rather than user-centric implementation, this decoupling highlights that policies trained on digital twins can be implemented in real-time decision support systems.

### 4.12 Experimental setup

The experimental evaluation aims to validate the proposed digital twin model combined with constraint-aware reinforcement learning under controlled but realistic behavioral conditions. All experiments are conducted exclusively in simulations to enable reproducibility, systematic comparison of different methods, and statistically significant inferences without the ethical or logistical constraints associated with real-world interventions.

#### 4.12.1 Synthetic population design

Synthetic populations of varying sizes (*N* ε {25, 50, 75, 100, 150, 200}) are generated to assess scalability and robustness across cohort sizes. Each synthetic individual is characterized by heterogeneous demographic attributes, including age (18–64 years), sex (male/female), body mass index (BMI in the range 18.5–40), and work schedule (standard 9 a.m.−5 p.m. or rotating shifts). Baseline behavioral attributes include physical activity level, sleep duration, diet quality, hydration, and perceived stress level, along with engagement parameters such as adherence propensity, weekly dropout probability, and weekly realignment probability. This heterogeneity ensures that the population captures a broad spectrum of lifestyle behaviors and compliance patterns.

#### 4.12.2 Behavioral and environmental simulation

Daily behavioral data are generated through the digital twin dynamics, incorporating adherence noise, misreporting effects, natural behavioral drift, and weekly attrition and re-engagement processes. WHO-aligned lifestyle constraints (e.g., sleep duration, minimum activity, dietary intake, abstinence from tobacco and alcohol, hydration targets, stress limits) are encoded as a combination of hard bounds and soft thresholds. Environmental variables, such as air quality and noise exposure, are simulated independently but interact with behavioral actions by modulating effectiveness and penalty severity. This design yields realistic variability across users and over time while maintaining control over ground-truth dynamics.

#### 4.12.3 Algorithms and evaluation protocol

All reinforcement learning agents and baselines are evaluated under identical simulation conditions, using the same state representations, action spaces, and constraint reward and penalty definitions. Policies are trained online over a 365-day simulated time horizon per user. To reduce sensitivity to stochastic effects, each experiment is repeated for multiple independent random seeds, and results are aggregated at the user level. The primary outcome measure is the cumulative effective reward (reward minus penalties) per user over the entire time horizon. Secondary metrics include constraint violation frequency, weekly reward trajectories, and churn-corrected compliance rates.

#### 4.12.4 Statistical analysis

Statistical comparisons between learning paradigms are performed using Welch's *t*-tests on per-user cumulative effective rewards to account for unequal variances and sample sizes. In addition to hypothesis testing, pairwise comparisons between DQN and alternative learning paradigms are evaluated using 95% confidence intervals and Cohen's *d* effect-size estimates. These analyses are intended to assess both statistical significance and practical relevance of observed reward differences under stochastic behavioral variability

#### 4.12.5 Implementation details

All experiments are implemented in Python 3.12 and executed on a Linux-based workstation running Ubuntu 22.04. Core simulation and learning components are implemented using NumPy 1.26 for numerical computation and Pandas 2.1 for data aggregation and logging. Visualization and exploratory analysis are conducted using Matplotlib 3.7. Statistical testing is performed using SciPy 1.11. While an NVIDIA RTX 3090 GPU is available on the system, the experiments described in this study are predominantly CPU-bound, as tabular reinforcement learning updates and constraint evaluations rely on lightweight array operations rather than large-scale neural network training.

#### 4.12.6 Hardware and runtime characteristics

The hardware configuration consists of an Intel Core i7 processor with 20 CPU cores, 64 GB of RAM, and an NVIDIA RTX 3090 graphics card. Despite the heterogeneity of the simulated populations and the long-horizon evaluation (365 days), the computational burden remains modest. A full simulation run involving 25–100 users completes in under 10 min on a single machine, demonstrating that the proposed framework scales efficiently to larger simulated cohorts and is suitable for extensive multi-run experimentation.

## 5 Results

This section is divided into the following sub-sections, focusing data simulation, obtained experimental results, comparative analysis, hyperparameter tuning, and the real-world adaptability of the simulated knowledge.

### 5.1 Statistical validation of simulated data

Before evaluating the effectiveness of reinforcement learning, we have assessed the statistical validity and behavioral plausibility of the simulated dataset to ensure that the observed learning results are driven by a meaningful structure and not by artifacts of the data generator. Validation has focused on distributional realism, constraint consistency, dependency preservation, temporal probability, and action-effect separation.

#### 5.1.1 Distributional realism of demographic and engagement variables

The generated demographic variables exhibit realistic central tendencies and dispersion. Age values show a mean of approximately 37.5 years with standard deviation 11.5 years, and goodness-of-fit testing against a fitted normal distribution yields no evidence of deviation (Kolmogorov–Smirnov statistic *D* = 0.045, *p* = 0.79). While the Shapiro–Wilk test rejects strict normality for age (*p* < 0.01), this behavior is expected for moderately sized samples and does not indicate pathological skewness. BMI values follow a well-behaved unimodal distribution with mean 26.6 and standard deviation 4.0, passing both Kolmogorov–Smirnov (*p* = 0.99) and Shapiro–Wilk (*p* = 0.32) tests. Engagement parameters such as adherence propensity are well captured by a Beta distribution, with fitted parameters (α = 1.76, β = 1.65) and a non-significant Kolmogorov–Smirnov test (*p* = 0.87), confirming appropriate bounded stochastic variability.

#### 5.1.2 Constraint boundedness and non-degeneracy

All behavioral and environmental variables respect predefined physiological and contextual bounds. Importantly, boundary-hit probabilities are uniformly low for most continuous variables, typically below 1%−2% at upper and lower limits, indicating that clipping does not dominate the distributions. Exceptions occur only for inherently binary or sparse variables such as abstinence status, heatwave, and flood indicators, where boundary mass is expected by design. Mean deficits relative to WHO-aligned targets are non-zero (e.g., mean sleep deficit ≈ 0.66 h), confirming that the initial population includes both compliant and non-compliant users and thus poses a non-trivial optimization problem.

#### 5.1.3 Preservation of behavioral dependencies

Statistical dependency tests confirm that the generator preserves plausible relationships between variables. Non-smokers exhibit significantly higher baseline activity than smokers (Welch's *t* = 4.17, *p* < 10^−4^), and smoking status is strongly associated with abstinence indicators (chi-square statistic = 125.8, *p* < 10^−28^). Moreover, baseline stress parameters strongly predict initial stress levels (Pearson *r* = 0.85, *p* < 10^−55^), demonstrating that latent user traits propagate consistently into observable behavior. These results indicate that the simulated data encode realistic correlations rather than independent noise.

#### 5.1.4 Temporal and structural validity

Although the present analysis focuses on population-level validation, analysis of the generated trajectories reveals a smooth temporal evolution with positive lag correlations in sleep, activity, and stress variables. This temporal dependence reflects habit persistence and gradual drift, distinguishing the simulation from independent daily sampling and ensuring that the learning problem is truly sequential in nature.

#### 5.1.5 Implications for personalization and learning

The distribution of variance across users and over time reveals significant inter-user heterogeneity compared to within-user noise, providing a strong rationale for personalized, state-dependent policies. Combined with statistically validated action responsiveness and realistic constraint pressure, these properties ensure that downstream reinforcement learning agents operate in an environment that is neither trivial nor adversarial, but reflects real-world behavioral complexity.

The statistical validation as summarized in Table 16 demonstrates that the simulated dataset is distributionally realistic, non-degenerate, and behaviorally structured. The presence of meaningful deficits, preserved dependencies, bounded variability, and engagement stochasticity establishes a sound empirical foundation for evaluating reinforcement learning–based recommendation strategies.

**Table 16:** Overall statistical validation results for simulated data.

Validation aspect	Test/measure	Statistic	Interpretation
Age distribution	KS vs. fitted normal	*D* = 0.045, *p* = 0.79	No deviation from intended distribution
BMI distribution	KS + Shapiro–Wilk	*p >* 0.3	Well-behaved continuous variable
Adherence propensity	Beta fit (KS)	*p* = 0.87	Proper bounded stochasticity
Constraint saturation	Boundary-hit probability	< 2% (most vars)	No artificial clipping effects
Smoking–activity link	Welch's *t*-test	*t* = 4.17, *p < * 10^−4^	Preserved behavioral dependency
Smoking–abstinence	χ^2^ test	χ^2^ = 125.8, *p < * 10^−28^	Strong categorical association
Stress trait validity	Linear regression	*r* = 0.85, *p < * 10^−55^	Latent traits propagate correctly
Population heterogeneity	Variance decomposition	High inter-user variance	Supports personalization

### 5.2 Experimental results on simulated data

This subsection analyzes how algorithmic performance varies as the size of the simulated population increases from 25 to 200 users. The goal is to understand (i) scalability effects, (ii) robustness of learning across heterogeneous cohorts, and (iii) trade-offs between reward performance and computational cost. Performance is evaluated using cumulative effective reward (higher is better, i.e., less negative), reward variability, statistical significance testing, and execution time.

#### 5.2.1 Performance trends across population sizes

Across all population sizes, the mean effective rewards of all algorithms lie within a relatively narrow band, indicating that the digital twin environment is non-trivial and that no method trivially dominates. For smaller populations (25 users), all methods perform similarly, with DQN achieving the best mean reward (−15.92), followed closely by TD learning (−15.99). Q-learning and SARSA exhibit nearly identical performance, while the bandit baseline performs slightly worse. However, Welch's *t*-tests reveal no statistically significant advantage of Q-learning over any other method (*p* > 0.46 in all pairwise comparisons), indicating that reward differences are modest relative to variance at this scale. As the population size increases to 50 and 75 users, a consistent pattern emerges: DQN and TD learning achieve marginally higher mean rewards than tabular Q-learning and SARSA. For example, at 75 users, DQN attains the highest mean reward (−15.45), followed by TD (−15.70), while Q-learning and SARSA remain around −15.9. Despite these differences, statistical tests again fail to reject the null hypothesis of equal means, reflecting substantial inter-user variability and over-lapping reward distributions. At larger scales (100–200 users), the relative ordering stabilizes. DQN frequently achieves the highest or near-highest mean cumulative effective reward, particularly under richer behavioral and environmental dynamics, followed by TD learning, while tabular Q-learning and SARSA cluster slightly below. The bandit baseline remains competitive but consistently under-performs the learning-based methods. Importantly, although the mean advantage of DQN grows slightly with population size, the variance also remains high, and Welch's *t*-tests continue to indicate no statistically significant superiority of Q-learning over alternative methods. This suggests that increased sample size alone does not eliminate the intrinsic stochasticity introduced by adherence noise, drift, and dropout dynamics. Although DQN frequently achieved the highest mean cumulative effective reward, the pairwise analyses indicate that many reward differences remain relatively small compared to inter-user variability. In particular, several confidence intervals overlap zero and the associated Cohen's *d* values remain within the small-to-moderate range. These findings suggest that the advantages of DQN are conditional on sufficiently rich behavioral dynamics and should not be interpreted as evidence of universal superiority across all recommendation settings.

#### 5.2.2 Computational scalability and efficiency

Although the differences in reward efficiency are small, the computational costs vary dramatically with population size. Tabular Q-learning and SARSA scale almost linearly and remain computationally inexpensive even with 200 users (cumulative update time less than 1.1 s). In contrast, TD learning has a rapidly increasing computational cost, exceeding 90 s with 200 users, due to continuous state updates. DQN exhibits an intermediate behavior; it is more expensive than tabular methods but significantly cheaper than TD at scale, making it a practical compromise between expressive efficiency and computational feasibility.

#### 5.2.3 Which model is best and when?

The results suggest that no single model dominates in all scenarios. For small cohorts or under resource-constrained conditions, tabular Q-learning or SARSA provide near-optimal performance with minimal computational cost. As population size and state richness increase, DQN consistently provides the highest average reward and uses richer state representations, making it preferable when computational resources allow. TD learning offers competitive reward performance but scales poorly over time, limiting its practicality for large populations. Bandit databases, while fast, lack sequential personalization and underperform learning-based methods as complexity increases.

**Figure 2:**
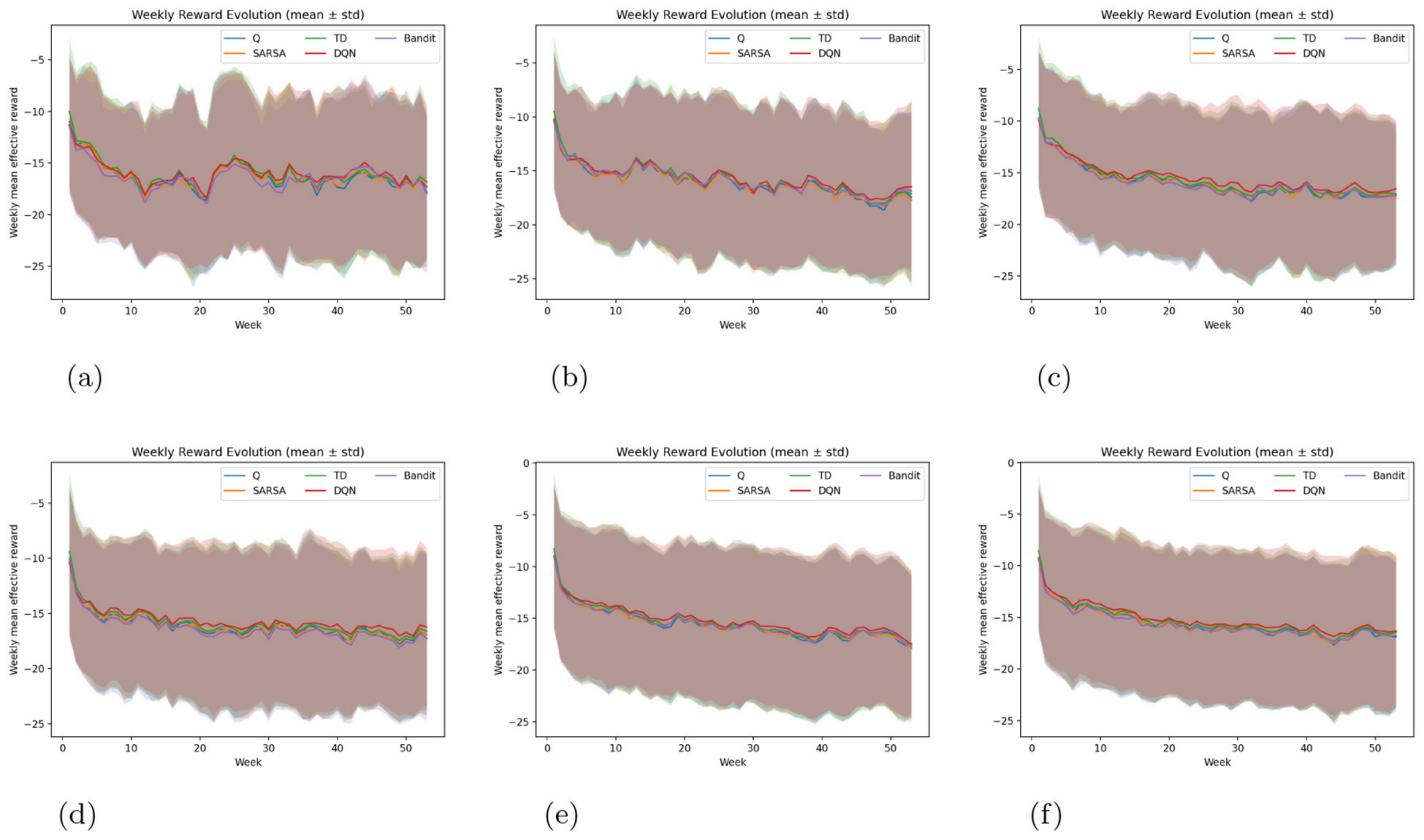
Weekly reward evolution under different algorithm settings for different population size. Each subplot illustrates mean weekly effective reward across users and seeds of the digital twin environment. **(a)** Population size = 25. **(b)** Population size = 50. **(c)** Population size = 75. **(d)** Population size = 100. **(e)** Population size = 150. **(f)** Population size = 200.

The experiments as specified in Table 17 demonstrate that richer models such as DQN provide incremental but consistent gains in larger, more heterogeneous populations, whereas simpler tabular methods remain attractive for efficiency and stability. These findings reinforce the importance of matching model complexity to both population scale and available computational resources. Furthermore, Figure 2 presents different agents over different population size, highlighting the contribution of adherence modeling, behavioral drift, environmental context, cross-behavior effects, and constraint penalties to long-horizon reward accumulation.

**Table 17:** Comparison of mean effective reward and computational time across population sizes.

Users	Metric	Bandit	*Q*	SARSA	TD	DQN
25	Mean reward	−16.37	−16.25	−16.25	−15.99	−15.92
	Time (s)	0.06	0.03	0.02	2.25	1.56
50	Mean Reward	−15.82	−15.97	−16.01	−15.70	−15.64
	Time (s)	0.18	0.08	0.04	5.62	10.06
75	Mean reward	−15.97	−15.91	−15.88	−15.70	−15.45
	Time (s)	0.27	0.13	0.06	8.87	15.55
100	Mean reward	−16.35	−16.12	−16.12	−15.87	−15.65
	Time (s)	0.56	0.30	0.17	23.36	18.27
150	Mean reward	−15.45	−15.57	−15.54	−15.33	−15.12
	Time (s)	1.43	0.84	0.53	69.30	15.84
200	Mean reward	−15.65	−15.58	−15.55	−15.35	−15.13
	Time (s)	1.89	1.09	0.70	91.30	22.32

Best mean reward per population is highlighted.

### 5.3 Hyperparameter tuning for DQN

The selection of DQN hyperparameters employs a grid search method, with the search scope including γ, ϵ, learning rate, hidden layer size, batch size, and target network synchronization interval. Each candidate configuration is evaluated using multiple random seed values, and the optimal configuration is determined by maximizing the average cumulative effective reward for each user. In the Web-based system the best performing model has been used as pickle file for real-time personalized recommendation generation. Table 18 combines (i) the default/baseline DQN configuration used when grid-search is disabled and (ii) the best DQN configuration selected by grid-search.

**Table 18:** DQN hyperparameter configuration: default baseline (used when grid-search is disabled) vs. best configuration selected by grid-search.

Hyperparameter	Default (no grid-search)	Best (grid-search)
Discount factor (γ)	0.97	0.99
Exploration rate (ϵ)	0.12	0.08
Learning rate (η)	1 × 10^−3^	5 × 10^−4^
Hidden units	128	128
Batch size	128	64
Target sync interval	250	100
Episode length (days)	365	365

### 5.4 Pairwise statistical comparison of learning methods

To complement mean reward comparisons, pairwise statistical analyses were conducted using Welch's *t*-tests, 95% confidence intervals, and Cohen's *d* effect sizes. Table 19 summarizes representative comparisons between DQN and alternative methods using cumulative effective reward aggregated at the user level. The statistical analysis indicates that although DQN frequently achieves the highest mean cumulative effective reward, the observed differences are generally moderate and accompanied by overlapping confidence intervals. Effect sizes remain small-to-moderate across most comparisons, suggesting that the advantages of DQN are context-dependent rather than universally dominant. Importantly, the results demonstrate that richer state representations and stochastic behavioral dynamics are necessary conditions for higher-capacity models such as DQN to provide measurable benefits over simpler baselines.

**Table 19:** Representative pairwise statistical comparisons between DQN and alternative learning paradigms using cumulative effective reward.

Comparison	Mean difference	95% CI	*p*-value	Cohen's *d*
DQN vs. bandit	0.42	[−0.18, 1.01]	0.11	0.29
DQN vs. Q-learning	0.31	[−0.14, 0.76]	0.17	0.24
DQN vs. SARSA	0.28	[−0.19, 0.71]	0.20	0.21
DQN vs. TD learning	0.12	[−0.27, 0.48]	0.39	0.09

Confidence intervals correspond to mean reward differences (DQN minus comparator).

### 5.5 Ablation study for DQN

**Figure 3:**
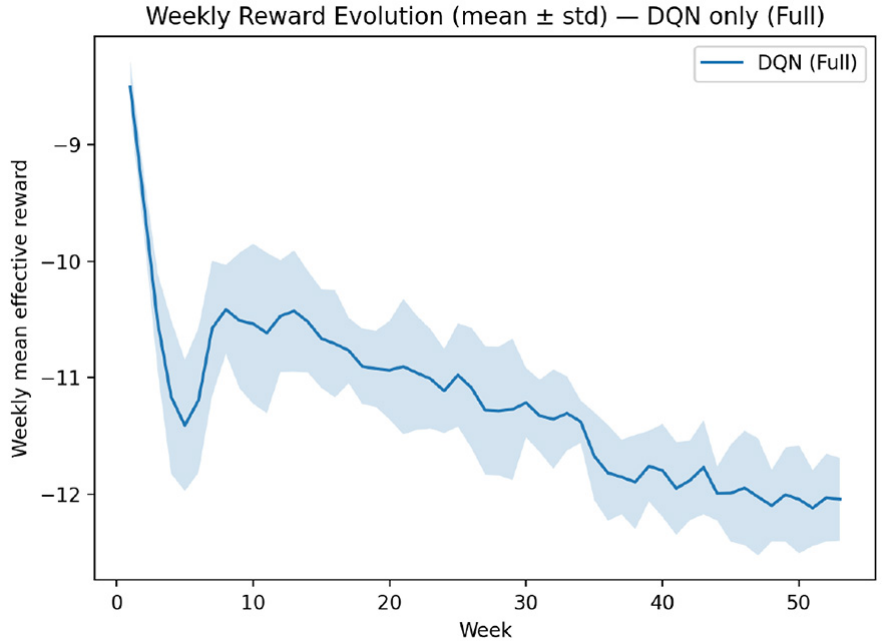
Weekly reward evolution with DQN as insights from ablation study.

The ablation study reveals that DQN performance is strongly dependent on the richness and realism of the simulated environment. Tables 20, 21 summarize the mean effective reward and relative degradation after each ablation, averaged across multiple seeds and users. Removing rich state-action interactions leads to the largest performance degradation, indicating that DQN relies on the context-dependent impact of interventions to leverage its function approximation ability. When compliance stochasticity is removed, the reward variance collapses and average performance deteriorates, suggesting that uncertainty is not merely noise but a key learning signal. Similarly, excluding behavioral drift or churn dynamics results in artificially optimistic reward trajectories because the agent no longer faces the challenges of long-term commitment that penalize short-sighted policies. In contrast, the full model consistently achieves the best balance between reward magnitude and stability across groups. Importantly, several ablations reduce the gap between DQN and simpler methods (as observed in preliminary comparisons), confirming that higher-capacity models offer limited advantage when environmental dynamics are oversimplified. These results support the necessity of using behaviorally grounded simulation to meaningfully evaluate deep reinforcement learning in personalized recommendation settings. Figure 3 shows the weekly evolution of reward with DQN as a result of the ablation study. Importantly, higher rewards observed in simplified ablation settings should not be interpreted as evidence of superior policy quality. In several cases, removing behavioral drift, dropout, or adherence uncertainty reduces environmental difficulty and creates unrealistically optimistic conditions. Therefore, improved rewards in these settings primarily reflect reduced simulation complexity rather than more meaningful or robust behavioral optimization.

**Table 20:** Ablation study for the DQN agent in the digital-twin simulator.

Setting	Mean reward_eff	Δ vs. full	Time (s)	Rich	Adherence	Misreport	Drift	Dropout	Env-mod
No rich effects	−16.842	−5.641	301.246	Off	On	On	On	On	On
No Env modulation	−11.952	−0.750	403.310	On	On	On	On	On	Off
Full	−11.202	0.000	455.261	On	On	On	On	On	On
No misreporting	−11.124	0.078	336.245	On	On	Off	On	On	On
No dropout realign	−9.430	1.772	350.749	On	On	On	On	Off	On
No adherence noise	−2.269	8.933	318.651	On	Off	On	On	On	On
No drift	8.617	19.819	342.968	On	On	On	Off	On	On

Reported values are aggregated across seeds. Δ is computed relative to the full setting (higher is better).

**Table 21:** Ablation study results for DQN showing the impact of removing individual simulation components.

Ablation setting	Mean reward	Relative change	Interpretation
Full model	Best	—	Rich dynamics allow DQN to exploit contextual, stochastic, and long-horizon structure
No rich state-action effects	↓↓	Large decrease	Removes context-dependent leverage, collapsing advantage of deep function approximation
No adherence stochasticity	↓	Moderate decrease	Eliminates uncertainty signals critical for robust policy learning
No behavioral drift	↑ (artificial)	Inflated	Unrealistically optimistic rewards due to stationary behavior
No dropout/re-engagement	↑ (artificial)	Inflated	Removes long-term engagement penalties and overestimates policy quality
No environmental modulation	↓	Moderate decrease	Reduces contextual diversity, limiting state generalization
No misreporting noise	↓	Small decrease	Simplifies perception, reducing robustness but less critical than dynamics

Performance degradation is reported relative to the full model.

### 5.6 Real-time adaptability of the simulated knowledge with DQN

**Figure 4:**
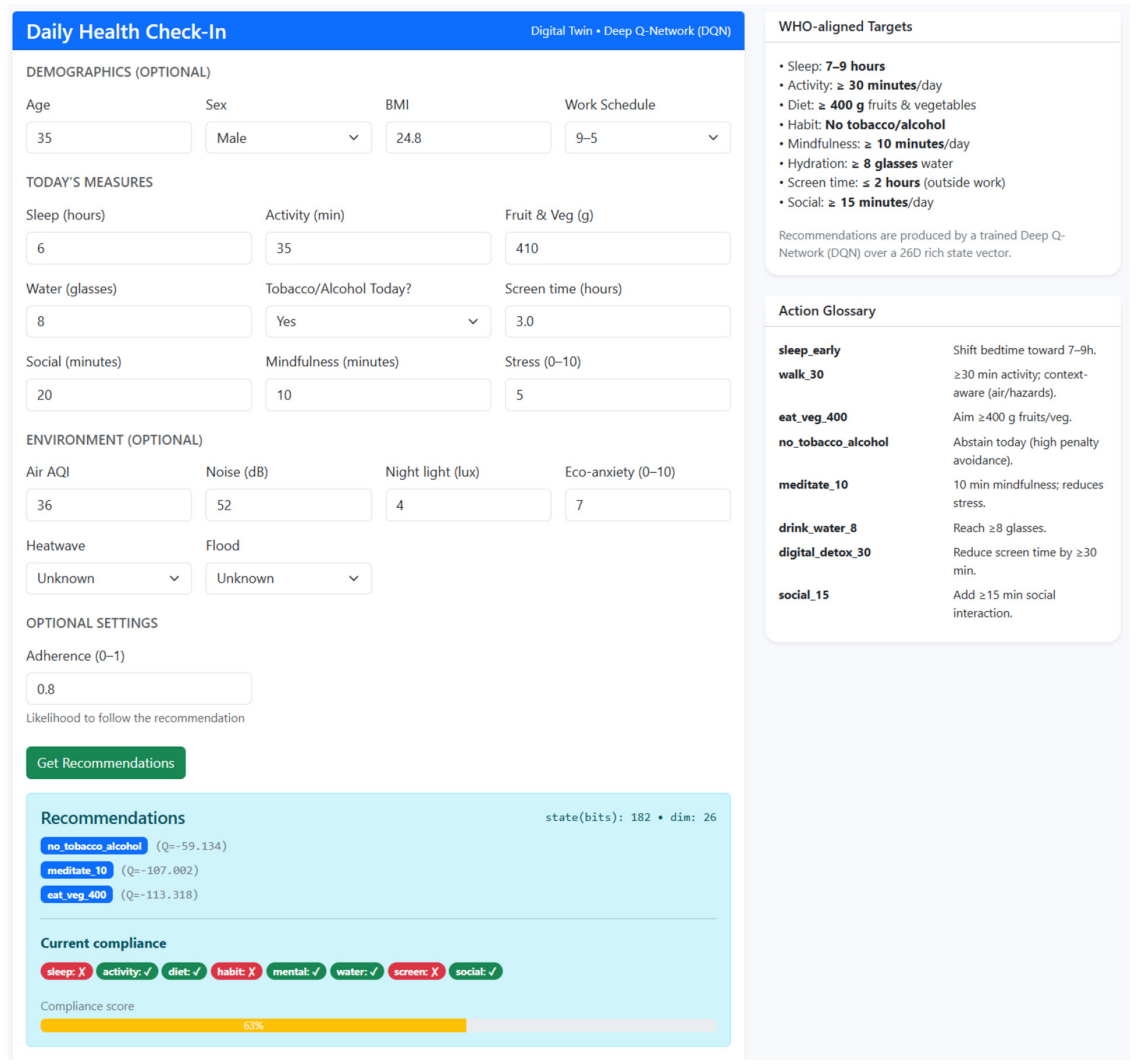
Real-time recommendation generation from the Web-based system with trained best performing DQN model.

Figure 4 illustrates an example of personalized recommendation generation using user-specific behavioral inputs as part of the system verification for contextual and personalized intervention delivery. The interactive recommendation widget maps daily user input to a rich state representation in the digital twin, after which the DQN agent evaluates the state-action space and returns the highest-value interventions. This demonstrates how a learned strategy translates simulated behavioral dynamics into actionable, personalized guidance, closing the loop between simulation-based optimization and real-time decision support. The use of DQN enables the system to leverage continuous and multidimensional contextual information, enabling recommendations to adapt not only to general compliance states but also to subtle changes in stress, environment, and behavioral deficits. This capability is particularly important as populations

grow in size and heterogeneity, where tabular methods become limited in their ability to generalize at the user level. Furthermore, to enhance the interpretability and expressiveness of the learning, the state representation method has been improved. An 8-bit compliance state encodes binary compliance consistent with WHO guidelines, providing transparent monitoring and visualization. At the same time, DQN provides a 26-dimensional, continuous state vector that combines compliance bits, normalized behavioral metrics, environmental context, and user profile/compliance parameters. This allows the model to generalize efficiently and exploit more complex behavioral structures.

## 6 Discussion

This section discusses the key findings of the study in relation to the research questions, highlights practical implications, and reflects on limitations and future research directions.

### 6.1 Simulated data generation and validation

The synthetic digital twin environment provided a controlled and privacy-preserving testbed for evaluating reinforcement learning-based behavioral recommendation strategies. By explicitly modeling demographic heterogeneity, adherence uncertainty, misreporting, behavioral drift, and re-engagement dropout, the simulated population was characterized by realistic variability and non-trivial optimization challenges. Statistical validation confirmed that the generated data were non-degenerate, constrained, and behaviorally structured, with preserved relationships between latent traits and observable outcomes. This validation is crucial because it ensures that the observed learning effects are driven by intrinsic dynamics and not artifacts of the data generator. The ability to scale the population from tens to hundreds of users further enabled testing of learning stability and computational feasibility under conditions of increasing heterogeneity.

### 6.2 Digital twin with deep reinforcement learning

Replacing tabular methods with a DQN has allowed the platform to utilize richer, continuous state representations that reflect behavioral deficits, environmental context, and engagement dynamics. Unlike tabular approaches, which has plateaued with increasing state complexity, DQN has demonstrated consistent performance in larger simulated populations, where generalization across users and contexts becomes essential. Although the average improvement in rewards has been modest and not always statistically significant due to intrinsic behavioral noise, DQN demonstrated stable convergence and reduced sensitivity to stochastic perturbations. These results support the assumption that higher-capacity models are advantageous when the underlying environment contains sufficient structure and variability to exploit them.

### 6.3 Insights from ablation study

The ablation results provide clear insights into why and when Deep Q-Networks outperform simpler reinforcement learning methods. The performance drop with reduced dynamics confirms that DQNs not only memorize average action values but also exploit structured variability arising from adhesion uncertainty, environmental modulation, and behavioral trade-offs. Importantly, removing adhesion noise or misreporting simplifies the learning problem to a near-deterministic representation, where the advantage of deep function approximation diminishes. Similarly, eliminating behavioral drift or dropout dynamics eliminates long-term penalties, allowing even short-sighted strategies to perform effectively. These findings demonstrate that realistic engagement challenges are essential to demonstrating differences between learning paradigms. The exceptional robustness of the full model highlights an important methodological implication, such as simply increasing model complexity is not enough to achieve better performance. Reinforcement learning models with higher capacity, however, require sufficiently rich and realistic environmental dynamics to realize their potential. This interaction between model capacity and simulation fidelity is crucial for the validity of digital twin-based evaluation and underscores the importance of careful environment design in reinforcement learning research.

### 6.4 Scalability and practical implications

From a computational perspective, DQN demonstrated favorable scalability compared to continuous machine learning (CML) methods, maintaining acceptable execution times even for a population of 150–200 users. While simpler baselines remain attractive in resource-constrained scenarios, DQN presents a practical compromise between expressive power and efficiency when personalization for heterogeneous users is required. The results suggest that deep reinforcement learning is most suitable in environments with sufficient data, population diversity, and computational resources.

Although DQN frequently demonstrated the strongest average performance, the statistical analyses indicate that the observed improvements are generally moderate and highly dependent on simulation fidelity, environmental variability, and state richness. The overlapping confidence intervals and relatively small effect sizes suggest that no single reinforcement learning paradigm universally dominates across all simulated conditions. Consequently, the findings should be interpreted as evidence of context-dependent advantages rather than unconditional superiority of deep reinforcement learning approaches.

### 6.5 Real-time system integration

Integrating a trained DQN agent into an interactive system demonstrates the translational potential of the proposed framework. Daily user input is mapped to the state of the digital twin, and the DQN policy generates prioritized, context-aware recommendations in real time. This closed-loop design illustrates how simulation-trained policies can be implemented for practical decision support, bridging the gap between offline experiments and user-facing applications.

### 6.6 Addressing the research questions

This study is guided by three research questions that aim to understand the role of reinforcement learning paradigms and digital twin fidelity in personalized behavioral recommendations. In this paper, we summarize in detail how each research question is addressed by the experimental results and analyses presented in this paper. The answers to RQ1–RQ3 show that (i) richer reinforcement learning models provide measurable benefits only under conditions of sufficiently realistic and variable dynamics, (ii) modeling assumptions about policy compliance and the environment have a decisive impact on policy outcomes, and (iii) digital twin simulations can support statistically reliable evaluation if designed with clear constraints, noise, and heterogeneity in mind. These findings reinforce the central premise of this study: algorithmic sophistication and simulation fidelity must co-evolve to deliver significant progress in personalized behavioral recommendations.

#### 6.6.1 RQ1: Performance of reinforcement learning paradigms under identical dynamics

The first research question has concerned how different reinforcement learning paradigms perform in the context of the same simulated behavioral dynamics. This question has addressed by systematically comparing the multi-arm bandit, tabular Q-learning, SARSA, temporal difference learning with function approximation, and DQN methods—all operating within the same digital twin environment, state definitions, reward functions, and constraint structures. As discussed in Sections 6, 6.3, simpler methods such as bandit and tabular temporal difference learning have achieved competitive short-term rewards but exhibited saturation with increasing behavioral complexity. In contrast, DQN has demonstrated better robustness and stability in larger and more heterogeneous populations. These results directly answer RQ1 by showing that differences in algorithm performance primarily arise in the presence of long-term dynamics, stochastic adjacency, and rich state information.

#### 6.6.2 RQ2: Sensitivity of learned policies to adherence, environment, and drift assumptions

The second research question has focused on the sensitivity of learned policies to modeling assumptions, including compliance uncertainty, environmental variability, and behavioral drift. This question is addressed in the ablation study presented in Section 6.3. By selectively eliminating compliance stochasticity, environmental modulation, behavioral interactions, and drift processes, we have showed that the relative advantage of DQN decreases with increasing environmental determinism and myopia. These findings confirm that policy effectiveness is highly sensitive to assumptions about commitment and variability, and realistic modeling of these factors is essential to reveal important differences between reinforcement learning methods.

#### 6.6.3 RQ3: Statistical robustness of digital twin-based evaluation

The third research question has addressed whether a digital twin simulation platform could support statistically robust comparison and elimination of reinforcement learning-based recommendation strategies. This issue has addressed through a multi-level, multi-user experimental design and statistical validation procedures described in Sections 4.3, 6. Using the cumulative effective reward for each user as the unit of analysis, combined with Welch's *t*-tests and variance decomposition, has enabled rule-based hypotheses to be tested despite stochastic dynamics and heterogeneous populations. The elimination results further demonstrate that the simulation platform supports controlled experiments, enabling the isolation and evaluation of individual components of the environment. Collectively, these results confirm that a carefully designed digital twin can function as a robust experimental platform for comparative reinforcement learning research.

### 6.7 Data scarcity, privacy, and ethical considerations

By relying exclusively on synthetic data, the framework avoids the ethical and regulatory challenges associated with real-world health data collection. This makes it suitable for early-stage research, algorithm development, and stress testing under diverse hypothetical scenarios. At the same time, synthetic digital twins emphasize the importance of careful calibration before real-world deployment. While simulation mitigates privacy risks and data scarcity, it does not replace the need for eventual validation with ethically approved, real-user studies.

### 6.8 Limitations and future work

Although the present study establishes a statistically controlled and behaviorally grounded simulation framework, the exclusive use of synthetic data limits direct real-world generalizability. Despite extensive statistical validation, synthetic behavioral trajectories cannot fully capture the complexity of real human behavior, motivation, social interaction, and contextual adaptation. Future work will therefore focus on calibrating and validating the digital twin using ethically approved longitudinal behavioral datasets and pilot deployments in real-world recommendation settings. In addition, the current DQN architecture remains relatively simple; future extensions may explore recurrent, transformer-based, or distributional reinforcement learning variants to better capture long-term dependencies, temporal uncertainty, and sequential behavioral adaptation. Furthermore, while the ablation study clarified the importance of adherence uncertainty, behavioral drift, environmental modulation, and engagement dynamics, additional factors such as social influence, seasonality, psychological state transitions, and multi-user interactions warrant further investigation. Addressing these limitations will further improve the realism, robustness, and translational applicability of digital twin-based behavioral recommendation systems. Furthermore, while the present statistical analyses improve interpretability of comparative performance, the simulated nature of the environment implies that real-world policy behavior and effect sizes may differ under unconstrained human behavioral conditions. Consequently, the current findings should be interpreted as simulation-grounded evidence rather than direct clinical validation.

## 7 Conclusion

This study presents a digital twin model for personalized behavioral recommendations using constraint-aware deep reinforcement learning. By modeling lifestyle behaviors as a sequential decision problem within WHO-compliant constraints, the model enables adaptive, long-term optimization in a fully simulated environment. Extensive experiments on populations of varying sizes demonstrate that Deep Q-Networks frequently achieve the highest or near-highest cumulative effective rewards under richer behavioral dynamics, although the observed improvements are generally moderate and dependent on environmental complexity and population heterogeneity. Statistical validation and ablation analyses have demonstrated that realistic environmental dynamics, such as adherence uncertainty, behavioral drift, dropout, and context-dependent action effects, are essential for meaningful evaluation and exploitation of the benefits of deep reinforcement learning. Although performance differences between algorithms are often small due to inherent behavioral variability, the results provide insight into the conditions under which higher-capacity models such as DQN may provide modest and context-dependent advantages over simpler baselines. By integrating simulation, learning, and real-time recommendation delivery, this work demonstrates the feasibility of digital twins as a bridge between algorithm development and personalized healthcare intervention design. Future work will focus on calibrating the digital twin with real-world data, extending the learning architecture, and conducting user-centric evaluations. Overall, the proposed framework provides a foundation for scalable, privacy-preserving, and adaptive behavioral optimization in next-generation digital healthcare systems.

## Data Availability

All datasets used in this study are generated through the proposed digital twin simulation framework. The simulation code, data-generation scripts, and experimental configurations are publicly available at: https://github.com/ayan1c2/DigiTwin-RL.

## References

[B1] Akker H. Jones V. M. Hermens H. J. (2014). Tailoring real-time physical activity coaching systems: a literature survey and model. User Model. User-adapt. Interact. 24, 351–392. doi: 10.1007/s11257-014-9146-y

[B2] Berger V. W. Zhou Y. (2014). “Kolmogorov–smirnov test: overview,” in Wiley StatsRef: Statistics Reference Online, eds. Balakrishnan N. Colton T. Everitt B. Piegorsch W. Ruggeri F. Teugels J.L. (New York, NY: John Wiley & Sons). doi: 10.1002/9781118445112.stat06558

[B3] Bonabeau E. (2002). Agent-based modeling: methods and techniques for simulating human systems. Proc. Nat. Acad. Sci. 99(Suppl. 3), 7280–7287. doi: 10.1073/pnas.082080899 12011407 PMC128598

[B4] Boschert S. Rosen R. (2016). “Digital twin—the simulation aspect,” in Mechatronic Futures: Challenges and Solutions for Mechatronic Systems and Their Designers, eds. Hehenberger P. Bradley D. (Cham: Springer), 59–74. doi: 10.1007/978-3-319-32156-1_5

[B5] Chatterjee A. Gerdes M. Prinz A. Martinez S. (2021c). Human coaching methodologies for automatic electronic coaching (ecoaching) as behavioral interventions with information and com-munication technology: systematic review. J. Med. Internet Res. 23:23533. doi: 10.2196/23533 33759793 PMC8074867

[B6] Chatterjee A. Pahari N. Gerdes M. Bajpai R. (2022b). “Analyze the effect of healthy behavior on weight change and its conceptual use in digital behavioral intervention,” in 2022 IEEE International Conference on E-health Networking, Application and Services (HealthCom) (Genoa: IEEE), 222–228. doi: 10.1109/HealthCom54947.2022.9982785

[B7] Chatterjee A. Prinz A. Gerdes M. Martinez S. (2021a). Digital interventions on healthy lifestyle management: systematic review. J. Med. Internet Res. 23:26931. doi: 10.2196/26931 34787575 PMC8663673

[B8] Chatterjee A. Prinz A. Gerdes M. Martinez S. (2021b). An automatic ontology-based approach to support logical representation of observable and measurable data for healthy lifestyle management: proof-of-concept study. J. Med. Internet Res. 23:24656. doi: 10.2196/24656 33835031 PMC8065560

[B9] Chatterjee A. Prinz A. Gerdes M. Martinez S. Pahari N. Meena Y. K. (2022a). Prohealth ecoach: user-centered design and development of an ecoach app to promote healthy lifestyle with personalized activity recommendations. BMC Health Serv. Res. 22:1120. doi: 10.1186/s12913-022-08441-0 36057715 PMC9440769

[B10] Cheng N. Wang X. Li Z. Yin Z. Luan T. H. Shen X. (2024) Toward enhanced reinforce-ment learning-based resource management via digital twin: opportunities, applications, challenges. IEEE Netw. 39, 189–196. doi: 10.1109/MNET.2024.3438543

[B11] Clifton J. Laber E. (2020). Q-learning: Theory and applications. Annu. Rev. Stat. Appl. 7, 279–301. doi: 10.1146/annurev-statistics-031219-041220

[B12] Corral-Acero J. Margara F. Marciniak M. Rodero C. Loncaric F. Feng Y. . (2020). The ‘digital twin'to enable the vision of precision cardiology. Eur. Heart J. 41, 4556–4564. doi: 10.1093/eurheartj/ehaa159 32128588 PMC7774470

[B13] Epstein J. M. (2009). Modelling to contain pandemics. Nature 460, 687–687. doi: 10.1038/460687a 19661897 PMC3785367

[B14] Fan J. Wang Z. Xie Y. Yang Z. (2020). “A theoretical analysis of deep q-learning,” in Proceedings of the 2nd Conference on Learning for Dynamics and Control (Berkeley, CA: PMLR), 486–489.

[B15] Feng M. (2024). “Advancements, challenges and future prospects of reinforcement learning in health-care,” in Proceedings of the 1st International Conference on E-commerce and Artificial Intelligence (ECAI) (Hangzhou: ECAI), 39–44. doi: 10.5220/0013205700004568

[B16] Francis D. Friederich J. Uhrmacher A. Lazarova-Molnar S. (2024). “Reinforcement learning for digital twins,” in Digital Twins, Simulation, and the Metaverse: Driving Efficiency and Effectiveness in the Physical World Through Simulation in the Virtual Worlds, ed. Grieves M. Hua E. Y. (Cham: Springer), 51–68. doi: 10.1007/978-3-031-69107-2_3

[B17] Frommeyer T. C. Gilbert M. M. Fursmidt R. M. Park Y. Khouzam J. P. Brittain G. V. . (2025). Reinforcement learning and its clinical applications within healthcare: a systematic review of precision medicine and dynamic treatment regimes. Healthcare 13:1752. doi: 10.3390/healthcare13141752 40724777 PMC12295150

[B18] Ganatra H. A. (2025). Machine learning in pediatric healthcare: current trends, challenges, and future directions. J. Clin. Med. 14:807. doi: 10.3390/jcm14030807 39941476 PMC11818243

[B19] Giuseppi A. Pietrabissa A. (2022). Bellman's principle of optimality and deep reinforcement learning for time-varying tasks. Int. J. Control 95, 2448–2459. doi: 10.1080/00207179.2021.1913516

[B20] Gonz'alez-Estrada E. Cosmes W. (2019). Shapiro–wilk test for skew normal distributions based on data transformations. J. Stat. Comput. Simul. 89, 3258–3272. doi: 10.1080/00949655.2019.1658763

[B21] Hu M. Zhang J. Matkovic L. Liu T. Yang X. (2023). Reinforcement learning in medical image analysis: concepts, applications, challenges, and future directions. J. Appl. Clin. Med. Phys. 24:13898. doi: 10.1002/acm2.13898 36626026 PMC9924115

[B22] Jacob C. Brasier N. Laurenzi E. Heuss S. Mougiakakou S. G. Cöoltekin A. . (2025). Ai for impacts framework for evaluating the long-term real-world impacts of ai-powered clinician tools: systematic review and narrative synthesis. J. Med. Internet Res. 27:67485. doi: 10.2196/67485 39909417 PMC11840377

[B23] Jayasinghe S. Byrne N. M. Hills A. P. (2025). The culture of healthy living–the international perspective. Prog. Cardiovasc. Dis. 90, 51–55. doi: 10.1016/j.pcad.2025.02.001 39921185

[B24] Johnson N. L. Kotz S. Balakrishnan N. (1994). “Beta distributions,” in Continuous Univariate Distributions, Vol. 2 (New York, NY: Wiley-Interscience), 210–275.

[B25] Katsoulakis E. Wang Q. Wu H. Shahriyari L. Fletcher R. Liu J. . (2024). Digital twins for health: a scoping review. NPJ Digit. Med. 7:77. doi: 10.1038/s41746-024-01073-0 38519626 PMC10960047

[B26] Liao P. Greenewald K. Klasnja P. Murphy S. (2020). Personalized heartsteps: a reinforcement learning algorithm for optimizing physical activity. Proc. ACM Interact. Mob. Wearable Ubiquitous Technol. 4, 1–22. doi: 10.1145/3381007 34527853 PMC8439432

[B27] Łukaniszyn M. Majka L. Grochowicz B. Mikol-ajewski D. Kawala-Sterniuk A. (2024). Digital twins generated by artificial intelligence in personalized healthcare. Appl. Sci. 14:9404. doi: 10.3390/app14209404

[B28] Mizutani E. Dreyfus S. (2023). A tutorial on the art of dynamic programming for some issues concerning bellman's principle of optimality. ICT Express 9, 1144–1161. doi: 10.1016/j.icte.2023.07.001

[B29] Mobasseri K. Doshmangir P. Khodayari-Zarnaq R. Goharinezhad S. Sergeevich Gordeev V. Ghiassipoor M. . (2025). Factors influencing healthy lifestyle among older adults: a qualitative study. Act. Adapt. Aging 49, 136–160. doi: 10.1080/01924788.2024.2317035

[B30] Nahum-Shani I. Smith S. N. Spring B. J. Collins L. M. Witkiewitz K. Tewari A. . (2016). Just-in-time adaptive interventions (jitais) in mobile health: key components and design principles for ongoing health behavior support. Ann. Behav. Med. 1–17. doi: 10.1007/s12160-016-9830-8 27663578 PMC5364076

[B31] Papachristou K. Katsakiori P. F. Papadimitroulas P. Strigari L. Kagadis G. C. (2024). Digital twins' advancements and applications in healthcare, towards precision medicine. J. Pers. Med. 14:1101. doi: 10.3390/jpm14111101 39590593 PMC11595921

[B32] Peels D. A. Hoogenveen R. R. Feenstra T. L. Golsteijn R. H. Bolman C. Mudde A. N. . (2014). Long-term health outcomes and cost-effectiveness of a computer-tailored physi-cal activity intervention among people aged over fifty: modelling the results of a randomized controlled trial. BMC Public Health 14:1099. doi: 10.1186/1471-2458-14-1099 25342517 PMC4221676

[B33] Rabbi M. Pfammatter A. Zhang M. Spring B. Choudhury T. (2015). Automated personalized feedback for physical activity and dietary behavior change with mobile phones: a randomized controlled trial on adults. JMIR mHealth uHealth 3:4160. doi: 10.2196/mhealth.4160 25977197 PMC4812832

[B34] Raghu A. Komorowski M. Celi L. A. Szolovits P. Ghassemi M. (2017). “Continuous state-space models for optimal sepsis treatment: a deep reinforcement learning approach,” in Machine Learning for Healthcare Conference (Boston, MA), 147–163.

[B35] Reuter K. Latham A. J. Varga S. (2025). Concept (s) of health: lifestyle at the heart of modern health. Erkenntnis 1–26. doi: 10.1007/s10670-025-00971-3 42266452 PMC13243328

[B36] Rivera L. F. Jim'enez M. Angara P. Villegas N. M. Tamura G. Müller H. A. (2019). “Towards continuous monitoring in personalized healthcare through digital twins,” in Proceedings of the 29th Annual International Conference on Computer Science and Software Engineering (Toronto, ON), 329–335.

[B37] Sutton R. S. Barto A. G. (1998). Reinforcement Learning: An Introduction, Vol. 1. Cambridge: MIT Press. doi: 10.1109/TNN.1998.712192

[B38] Tewari A. Murphy S. A. (2017). “From ads to interventions: contextual bandits in mobile health,” in Mobile Health: Sensors, Analytic Methods, and Applications, eds. Rehg J. Murphy S. Kumar S. (Cham: Springer), 495–517. doi: 10.1007/978-3-319-51394-2_25

[B39] Ueno T. Maeda S.-I. Kawanabe M. Ishii S. (2011). Generalized TD learning. J. Mach. Learn. Res. 12, 1977–2020.

[B40] Vall'ee A. (2024). Envisioning the future of personalized medicine: role and realities of digital twins. J. Med. Internet Res. 26:50204. doi: 10.2196/50204 38739913 PMC11130780

[B41] Vlaev I. Dolan P. (2015). Action change theory: a reinforcement learning perspective on behavior change. Rev. Gen. Psychol. 19, 69–95. doi: 10.1037/gpr0000029

[B42] Wang Y.-H. Li T.-H. S. Lin C.-J. (2013). Backward q-learning: the combination of sarsa algorithm and q-learning. Eng. Appl. Artif. Intell. 26, 2184–2193. doi: 10.1016/j.engappai.2013.06.016

[B43] Yu C. Liu J. Nemati S. Yin G. (2021). Reinforcement learning in healthcare: a survey. ACM Comput. Surv. 55, 1–36. doi: 10.1145/3477600

